# Plasmacytoid dendritic cells and RNA-containing immune complexes drive expansion of peripheral B cell subsets with an SLE-like phenotype

**DOI:** 10.1371/journal.pone.0183946

**Published:** 2017-08-28

**Authors:** Olof Berggren, Niklas Hagberg, Andrei Alexsson, Gert Weber, Lars Rönnblom, Maija-Leena Eloranta

**Affiliations:** 1 Department of Medical Sciences, Rheumatology, Science for Life Laboratory, Uppsala University, Uppsala, Sweden; 2 Department of Molecular Structural Biology, Institute of Biochemistry, Ernst-Moritz-Arndt University of Greifswald, Greifswald, Germany; Instituto Nacional de Ciencias Medicas y Nutricion Salvador Zubiran, MEXICO

## Abstract

**Background:**

Hyperactive B cells and a continuous interferon (IFN)-α production by plasmacytoid dendritic cells (pDCs) play a key role in systemic lupus erythematosus (SLE). We asked whether the interaction between B cells and pDCs stimulated with RNA-containing immune complexes affects peripheral B cell subsets.

**Methods:**

B cells and pDCs were isolated from blood of healthy individuals and stimulated with immune complexes consisting of SLE-IgG and U1snRNP (RNA-IC). Expression of cell surface molecules as well as IL-6 and IL-10 production were determined by flow cytometry and immunoassays. Gene expression profiles were determined by a NanoString nCounter expression array.

**Results:**

We found a remarkable increase of double negative CD27^-^IgD^-^ B cells, from 7% within fresh CD19+ B cells to 37% in the RNA-IC-stimulated co-cultures of B cells and pDCs, comparable to the frequency of double negative B cells in SLE patients. Gene expression analysis of the double negative CD27^-^IgD^-^ and the CD27^+^IgD^-^ memory B cells revealed that twenty-one genes were differentially expressed between the two B cell subsets (≥ 2-fold, p<0.001). The, *IL21R*, *IL4R*, *CCL4*, *CCL3*, *CD83* and the *IKAROS Family Zinc Finger 2 (IKZ2)* showed higher expression in the double negative CD27^-^IgD^-^ B cells.

**Conclusion:**

The interactions between B cells and pDCs together with RNA-containing IC led to an expansion of B cells with similar phenotype as seen in SLE, suggesting that the pDC-B cell crosstalk contributes to the autoimmune feed-forward loop in SLE.

## Introduction

Hyperactivated B cells, autoantibodies to nuclear components and an activated type I interferon (IFN) system are common features in patients with systemic lupus erythematosus (SLE) [1–3]. Accordingly, alterations of B cell subsets have been widely documented in SLE, e.g. an expansion of isotype switched CD27^+^IgD^-^ memory B cells, plasma cells and double negative CD27^-^IgD^-^ B cells [1, 4, 5]. The CD27^-^IgD^-^ B cells are particularly increased in patients during disease flares and are associated with presence of nephritis, autoantibodies to dsDNA and RNP/Smith autoantigens [6, 7]. Despite the lack of CD27 expression, the CD27^-^IgD^-^ B cell subset contains a fraction of switched memory B cells expressing CD95, indicating an activated phenotype [7, 8]. In addition to antibody production, B cells have other important regulatory functions and influence immune responses by cytokine production, autoantigen presentation and co-stimulation of T cells [4, 9, 10]. Lately, the impact of regulatory B cells (Bregs), functionally characterized by their IL-10 and TGF-β production, has been highlighted in etiopathogenesis of autoimmune diseases, including SLE [11–13].

Type I IFN is a fundamental mediator of SLE immunopathology and has well documented effects on B cells, such as increase of plasma cell differentiation, survival and Ig isotype switch [3, 14]. Despite this, rather little is known how different B cell subsets are affected by interactions with the professional IFN-α producers i.e. plasmacytoid dendritic cells (pDCs) [15]. Recently it was shown that pDCs stimulated with CpG DNA promote the development of functionally competent CD24^hi^CD38^hi^ Bregs cells from healthy individuals in an IFN-α dependent manner. Furthermore, this pathway was impaired in patients with SLE, leading to defect immunosuppressive capacity of the Breg cells [13, 16].

Although a number of B cell disturbances are recognized in SLE and other autoimmune diseases, the mechanism behind the generation of hyperreactive B cells and loss of tolerance is not fully clarified i.e. whether it is an intrinsic attribute of B cells or a consequence of the environmental conditions. We have previously demonstrated a functionally important cellular cross-talk, where B cells enhanced the IFN-α production by pDCs activated with IC consisting of U1 snRNP particles and autoantibodies from SLE patients [17]. In the current study we therefore explored the development of several SLE associated B cell subsets after exposure of peripheral B cells from healthy individuals to RNA-containing IC and pDCs.

## Materials and methods

### Patients and healthy donors

All patients (n = 12) fulfilled ≥ 4 of the American College of Rheumatology classification criteria for SLE [18]. The blood samples were collected during ordinary visits at the outpatient Rheumatology clinic at Uppsala University Hospital. Healthy blood donors were recruited at the Department of Transfusion Medicine, Uppsala University Hospital. The regional ethics committee of Uppsala approved the present study (approval numbers: Dnr2009/013 and Dnr2016/155). All patients and healthy individuals gave their written informed consent.

### Cell isolation and culture conditions

Peripheral blood mononuclear cells (PBMCs) were purified from healthy blood donor buffy coats or EDTA-blood from SLE patients and healthy donors using Ficoll-Hypaque (GE Healthcare, Uppsala, Sweden) density-gradient centrifugation. PDCs and B cells were isolated from PBMCs by magnetic bead separation using negative selection (pDC Isolation kit and B cell Isolation kit II; Miltenyi Biotec, Bergisch Gladbach, Germany). Purity of the isolated cell populations was determined by flow cytometry after staining with anti-BDCA2 (Miltenyi Biotec) or anti-CD19 (BD Biosciences) monoclonal antibodies (mAbs) and was found to be at least 95%. The pDCs and B cells were cultured as previously described with 0.25x10^5^ pDCs and 1x10^5^ B cells in 0.1 ml volumes per well in 96-well plates for 20 hours or 4 to 6 days as indicated, at 37°C with 5% CO_2_ [17]. When indicated, affinity purified polyclonal goat anti-BAFF (#AF124) or normal goat IgG antibodies (R&D Systems) were added to the co-cultures of pDCs and B cells at a final concentration of 20 μg/ml at the beginning of the cell culturing.

#### Interferon inducers

U1 snRNP particles were purified from HeLa cells as described before [19] and SLE-IgG was isolated from two SLE patient sera containing autoantibodies to Sm and RNP autoantigens, by protein G chromatography. The U1 snRNP particles and SLE-IgG were used in cell cultures at final concentrations of 2.5 μg/ml and 1 mg/ml, respectively. All cultures were supplemented with IL-3 (1 ng/ml) and GM-CSF (4 ng/ml) in order to sustain the cell viability of pDCs [20, 21]. The cell viability of the B cells and pDCs in the 6 day cultures was determined by using LIVE/DEAD near-IR dead cell stain (Molecular Probes, Eugene, OR) and was found to be at least 65–70% of the single cells.

### Flow cytometry

Both fresh and *in vitro* cultured B cells and pDCs were stained with fluorochrome labeled mAbs (BD Biosciences) for flow cytometric analysis. B cells and pDC were identified by CD19 (clone: HIB19) and CD123 (7G3) expression, respectively (S1 Fig). The B cells were further characterized by staining with mAbs to CD27 (M-T271), CD38 (HIT2), IgD (IA6-2), CD24 (ML5) and CD27 (M-T271). In addition, the B cells were characterized as CD27^+^IgD^-^ switched memory cells (SM), CD27^+^IgD^+^ non-switched memory cells (NSM), CD27^-^IgD^+^ naive cells (N), CD27^hi^IgD^-^ plasmablasts (PC) and CD27^-^IgD^-^ double negative B cells (DN) [22]. Activation level of the B cells was analyzed by mAbs to CD80 (L307.4), CD86 (FUN-1) and CD95 (DX2). Isotype matched irrelevant mAbs were used as controls. The antibodies were purchased from BD Biosciences or Miltenyi Biotec. The intracellular detection of cytokines was performed after 6 days of culturing, the last 5 h in presence of brefeldin A (10 μg/ml). The cells were fixed with 2% paraformaldehyde/PBS and stained with PE-conjugated anti-IL-6 or APC-conjugated anti-IL-10 mAbs (R&D Systems), or corresponding isotype controls, in PBS with 0.5% saponin. The stained cells were analyzed by a FACSCantoII flow cytometer (BD Biosciences) and FlowJo VX (Tree Star, Ashland, OR) software. The cells were gated as singlets, and as live cells using LIVE/DEAD near-IR dead cell stain (Molecular Probes) before further analysis.

For gene expression analysis the B cells were cultivated with pDCs and RNA-IC for 4 days and sorted by FACSAria III (BD Biosciences) after staining with monoclonal antibodies to CD123, CD19, CD27 and IgD. Two B cell fractions were isolated from each cell donor based on CD19^+^CD27^-^IgD^-^ or CD19^+^CD27^+^IgD^-^ phenotype. Cell sorting was performed at +4°C and the collected cells were lysed in RLT-buffer (Qiagen) with 0.1% β-mercaptoethanol before frozen at -80°C.

### Gene expression profiling

Gene expression profile of the sorted CD27^-^IgD^-^ and CD27^+^IgD^-^ B cell subsets from 10 individuals were analyzed by using Human Immunology V2 expression array kit profiling 594 immunology-related genes and a Custom CodeSet consisting of 20 additional genes (NanoString Technologies, Seattle, WA) (S1 Table). Briefly, the B cell lysates (10 000 cells/5 μl) were hybridized at 65°C for 18–20 hours, posthybridized immediately on the nCounter Prep Station and the signals were quantified with nCounter Digital Analyzer (NanoString nCounter platform). The background level was defined by the counts generated with the negative control probes (geometric mean +3 standard deviations). The raw counts were normalized by using internal controls and housekeeping genes (nSolver v3.0 software (NanoString). A threshold for positive expression signal was set ≥ 50 counts. The normalized counts were log10 transformed before further analysis. To control the reproducibility of the nCounter expression array ten samples were run as technical replicates, and generated similar results (not shown).

### Immunoassays

IFN-α was analyzed in culture supernatants after 20 h and 6 days by a dissociation-enhanced lanthanide fluoroimmunoassay (DELFIA) [23]. IL-6, IL-10 and TGF-β in the culture supernatants were analyzed by using the CBA Human Soluble Protein Flex Set system (BD Biosciences) while BAFF was analyzed by an ELISA (R&D systems).

### Statistics

Graph Pad Prism Software 6.0 (GraphPad Software, La Jolla, CA) and SciPy 0.18.0 (Open source scientific tools for Python, http://www.scipy.org/) were used for statistical analysis. Differences between groups were analyzed by Wilcoxon signed rank test, Mann-Whitney U test or Friedmans test with Dunn’s correction for multiple comparisons. P values ≤ 0.05 were considered statistically significant when comparing cell frequencies and cytokine levels between groups. A significance level of p< 0.001 was applied on the gene expression analysis data.

## Results

### Double negative CD27^-^IgD^-^ B cells expand in the presence of RNA-IC and pDCs

**Fig 1:**
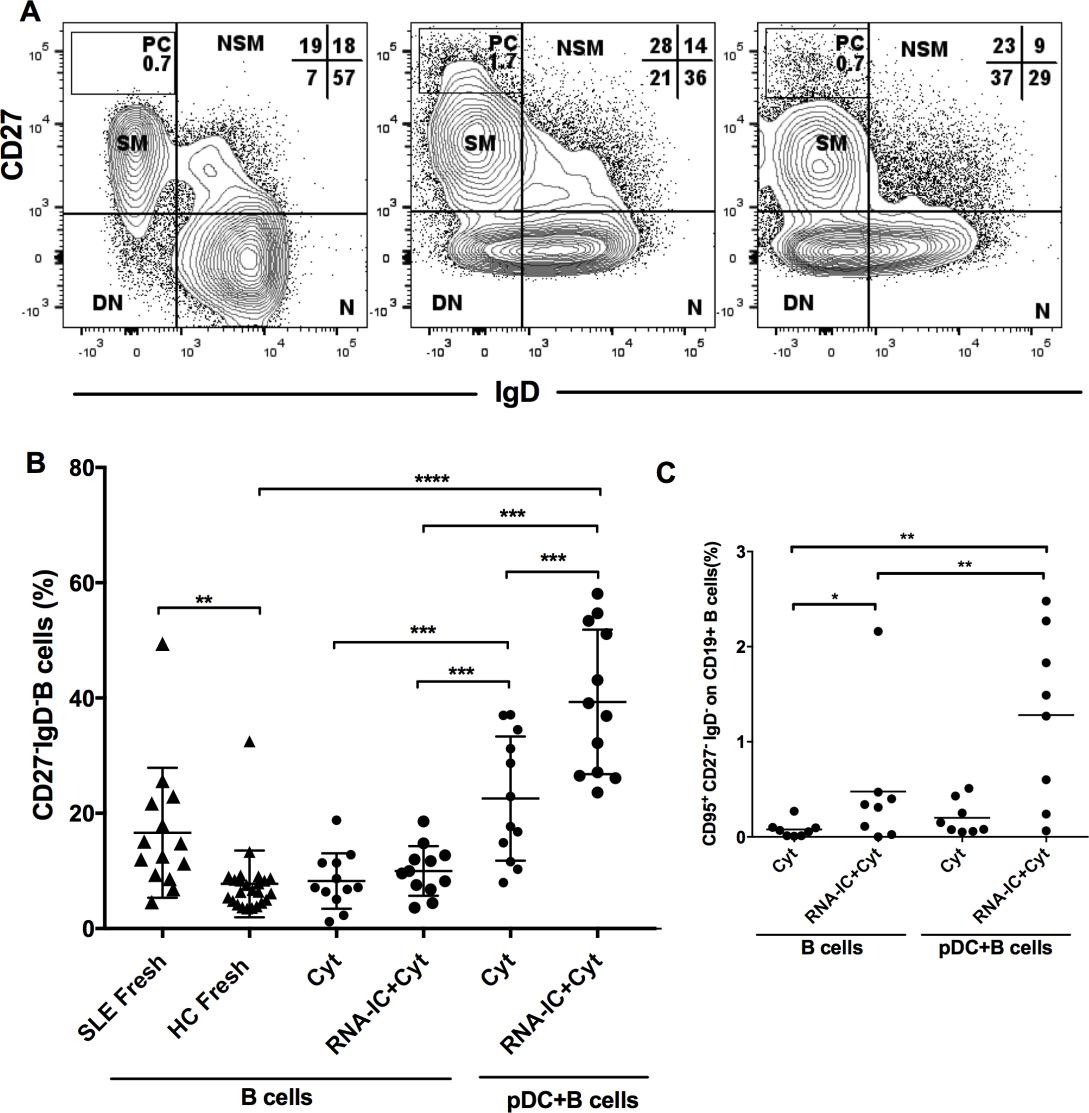
Plasmacytoid dendritic cells and RNA-containing immune complexes increase the frequency of CD27^-^IgD^-^ B cells. Flow cytometric analysis of peripheral blood B cells isolated from SLE patients (SLE) or from healthy individuals (HC). The CD19^+^ B cells were stained without culturing (fresh) or either cultured alone or together with plasmacytoid dendritic cells (pDC) for 6 days, in absence or presence of RNA-containing immune complexes (RNA-IC). All cultures were supplemented with IL-3 and GM-CSF (Cyt). (A) Representative plots and gating strategy of B cells stained for the cell surface expression of CD27 and IgD, and categorized as: CD27^+^IgD^-^ switched memory cells (SM), CD27^hi^IgD^-^ plasmablasts (PC), CD27^+^IgD^+^ non-switched memory cells (NSM), CD27^-^IgD^+^ naive cells (N) and CD27^-^IgD^-^ double negative B cells (DN). The plots show B cells fom healthy individuals stained without culturing (left plot), cultured in presence of IL-3/GM-CSF (middle plot), or stimulated with RNA-IC+ IL-3/GM-CSF (right plot). (B) The frequency of CD27^-^IgD^-^ B cells in the total CD19^+^ B cell population. (C) The frequency of CD27^-^IgD^-^CD95^+^ B cells in the total CD19^+^ B cell population. (B, C) Individual values (dots) and the mean (horizontal bars) are shown. * = p<0.05, ** = p<0.01, *** = p<0.001, **** = p<0.0001. Statistical analyses were performed by Mann Whitney test or Wilcoxon signed rank test.

Initially, we asked if pDCs and RNA-containing IC could facilitate the expansion of double negative CD27^-^IgD^-^ B cells to the same extent as seen in patients with SLE. Therefore, healthy donor B cells were cultured, alone or together with pDCs, in presence or absence of RNA-IC. After 6 days, the B cells were characterized based on the expression of CD19, CD27 and IgD by flow cytometry [22] and the results were compared with peripheral B cells from patients with SLE. Fig 1A shows representative gating of freshly isolated B cells from a healthy donor (left panel) or B cells co-cultured with pDCs, in absence (middle panel) or presence of RNA-IC (right panel).

Patients with SLE had an increased proportion of double negative CD27^-^IgD^-^ B cells among peripheral CD19^+^ B cells (16 ± 5.8%, mean ± SD) compared to healthy individuals (7.8 ± 5.8%, p<0.01) (Fig 1B). Strikingly, the frequency of double negative CD27^-^IgD^-^ B cells among the healthy donor B cells increased to 22 ± 11%, (p = 0.0001) when cultured together with pDCs, and showed an additional increase in the RNA-IC-stimulated co-cultures (39 ± 13%, p<0.0001). There was no expansion of double negative B cells in cultures without pDCs. Thus, co-cultivation of B cells and pDCs stimulated an increase of the CD27^-^IgD^-^ B cells that was further potentiated by RNA-IC.

The CD95 receptor is expressed on the memory fraction of double negative CD27^-^IgD^-^ B cells and is suggested to indicate an active phenotype. It is also associated with SLE disease flares [24]. Therefore, we examined the expression of CD95 on B cells after 6 days culturing. We found the frequency of CD95 on the double negative B cells to be significantly higher when B cells were stimulated with RNA-IC (0.48±0.70%, mean ± SD, p<0.05) compared to GM-CSF/IL-3 only (0.077 ± 0.086%), and there was an almost threefold additional increase in the presence of pDCs (1.28 ± 0.9%, p<0.01) (Fig 1C). Thus, when healthy blood donor B cells were exposed to RNA- containing IC together with pDCs, the double negative CD27^-^IgD^-^ B cell subset with similar phenotype as present in SLE was expanded.

Next, we asked if the increased frequency of the double negative CD27^-^IgD^-^ B cells was due to produced IFN-α, but could not detect any effect when neutralizing antibodies to IFN-α were added into RNA-IC stimulated pDC/B cell co-cultures (not shown).

**Fig 2:**
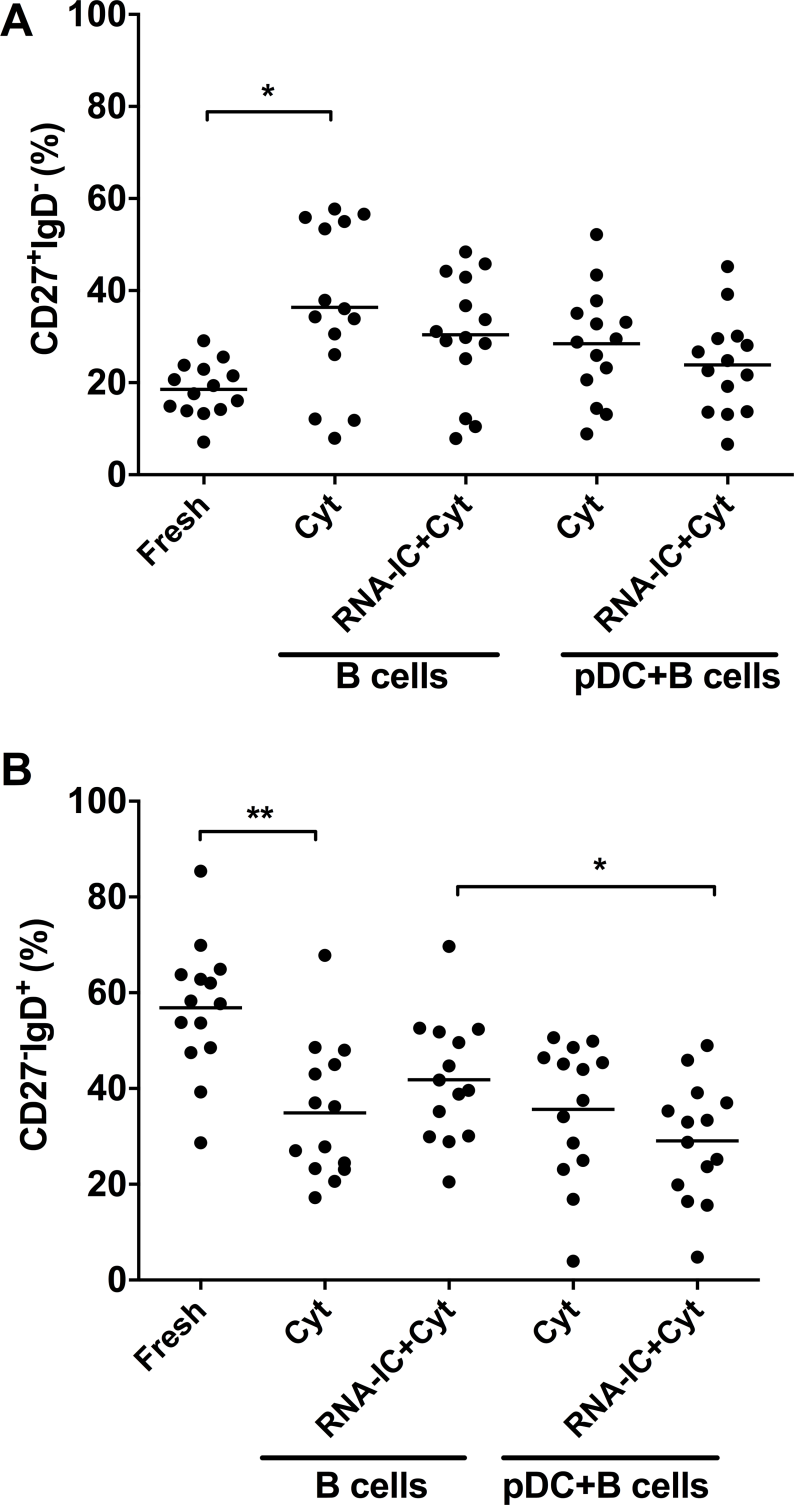
Frequency of switched memory and naïve B cells in presence of plasmacytoid dendritic cells and RNA-containing immune complexes. Flow cytometric analysis of B cells, without culturing (fresh) or stimulated for 6 days with IL-3/GM-CSF (Cyt) in absence or presence of RNA-containing immune complexes (RNA-IC). The CD19^+^ B cells were either stimulated alone or in co-cultures with plasmacytoid dendritic cells (pDC). (A) The percentage of CD27^+^IgD^-^ isotype switched memory (SM) B cells. (B) The percentage of CD27^-^IgD^+^ naïve B cells. Individual values (dots) and mean values (horizontal bars) are shown * = p<0.05, ** = p<0.01. Statistical analyses were performed by Friedman test.

We also investigated the proportion of antigen switched memory (SM) CD27^+^IgD^-^ B cells [22] in the pDC/B cell co-cultures, because these cells have been reported to be increased in patients with SLE (1). When analyzing the frequency of CD27^+^IgD^-^ B cells we found an increase from 19 ± 6% among fresh B cells to 36 ± 18% (mean ± SD, p< 0.05) in the cultures with B cells stimulated with IL-3/GM-CSF (Fig 2A). No increase of the switched memory CD27^+^IgD^-^ B cells was triggered by co-cultivation with pDCs or RNA-IC.

In contrast, the proportion of the CD27^-^IgD^+^ naïve B cells (N) (57± 14%, mean ±SD) was decreased upon cultivation with IL-3/GM-CSF (35 ± 14%, mean ±SD, p<0.01) (Fig 2B). Again no additional effect was achieved by stimulation with RNA-IC while in the presence of activated pDCs a slight decrease of naive B cells was noted (p<0.05). Consequently, the pDCs and RNA-IC did not contribute to any substantial change in the proportion of switched memory B cells or naïve B cells.

In summary, interaction between healthy donor B cells and pDCs in the presence of RNA-containing IC triggers a marked expansion of double negative CD27^-^IgD^-^ B cells that normally are low in healthy individuals but can be a predominant peripheral B cell subset in SLE.

### RNA-containing immune complexes and pDCs have activating effect on IL-6 and IL-10 production by B cells

Next, we asked whether a microenvironment comprising of pDCs and RNA-containing IC could activate expression of immunomodulatory surface molecules and cytokines with the capacity to affect B cell function. Consequently, B cells and pDCs were cultured together for six days in the presence or absence of RNA-IC. Subsequently, expression of the co-stimulatory molecules CD86 and CD80 were determined on B cells whereas pDCs and B cells were analyzed for intracellular IL-6 and IL-10. The cell culture supernatants were analyzed for the cytokines IL-6, IL-10, IFN-α, TGF-β and BAFF.

**Fig 3:**
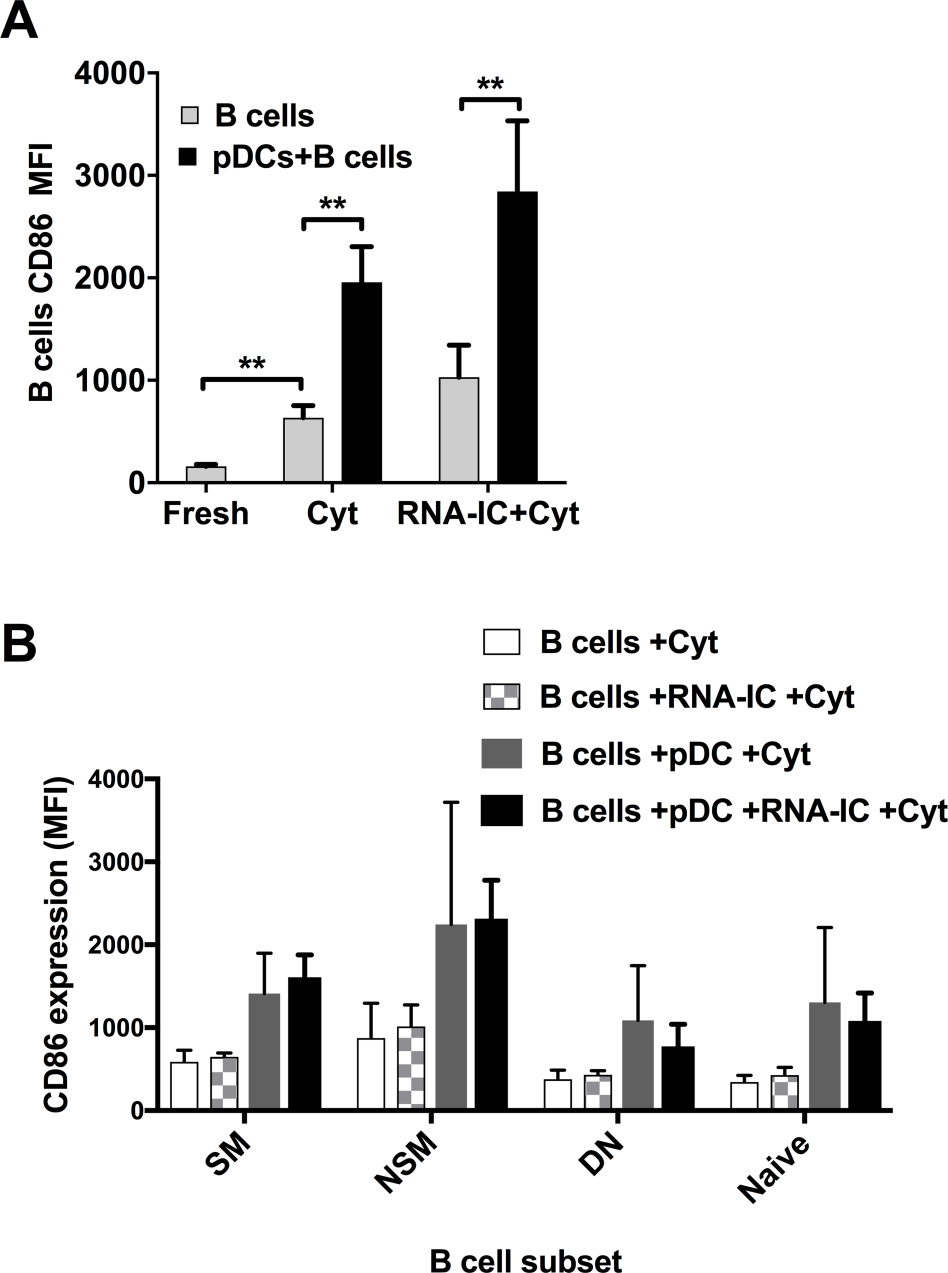
CD86 expression by B cells cultured with plasmacytoid dendritic cells and RNA-containing immune complexes. Flow cytometric analysis of CD86 expression by (A) CD19+ B cells and (B) subsets of B cells. A) The B cells were analyzed without culturing (fresh) or after culturing for 6 days with IL-3/GM-CSF (Cyt), in absence or presence of RNA-containing immune complexes (RNA-IC) and plasmacytoid dendritic cells (pDCs). (B) The CD86 expression was further analyzed on B cell subsets characterized as switched memory cells (CD27^+^IgD^-^), non-switched memory (CD27^+^IgD^+^), double negative (CD27^-^IgD^-^) or naïve (CD27^-^IgD^+^) B cells. The median fluorescence intensity (MFI) of CD86 expression is based on (A) 9 and (B) 4–6 individual donors, respectively. * = p<0.05, ** = p<0.01. Statistical analyses were performed by Wilcoxon signed rank test.

The expression of CD86 by B cells was significantly higher in the co-cultures with pDCs compared to cultures with only B cells (p< 0.01) (Fig 3A), whereas CD80 expression on B cells was not changed in the co-cultures (not shown). In addition, we analyzed the CD86 expression on the B cells subsets classified as switched memory (CD27^+^IgD^-^), non-switched memory (CD27^+^IgD^+^), double negative (CD27^-^IgD^-^) and naïve B cells [22]. The CD86 expression by the different B cell subsets demonstrated the same pattern as the whole CD19+ B cell population i.e. the strongest expression of CD86 was present on the B cells co-cultivated with pDCs (Fig 3B). Furthermore, the non-switched CD27^+^IgD^+^ memory B cells appeared to express higher levels of CD86 than the other B cell subsets. Especially in the RNA-IC-stimulated co-cultures the CD86 expression of the CD27^+^IgD^+^ B cells was significantly higher in comparison with the double negative CD27^-^IgD^-^ B cells (p<0.05).

**Fig 4:**
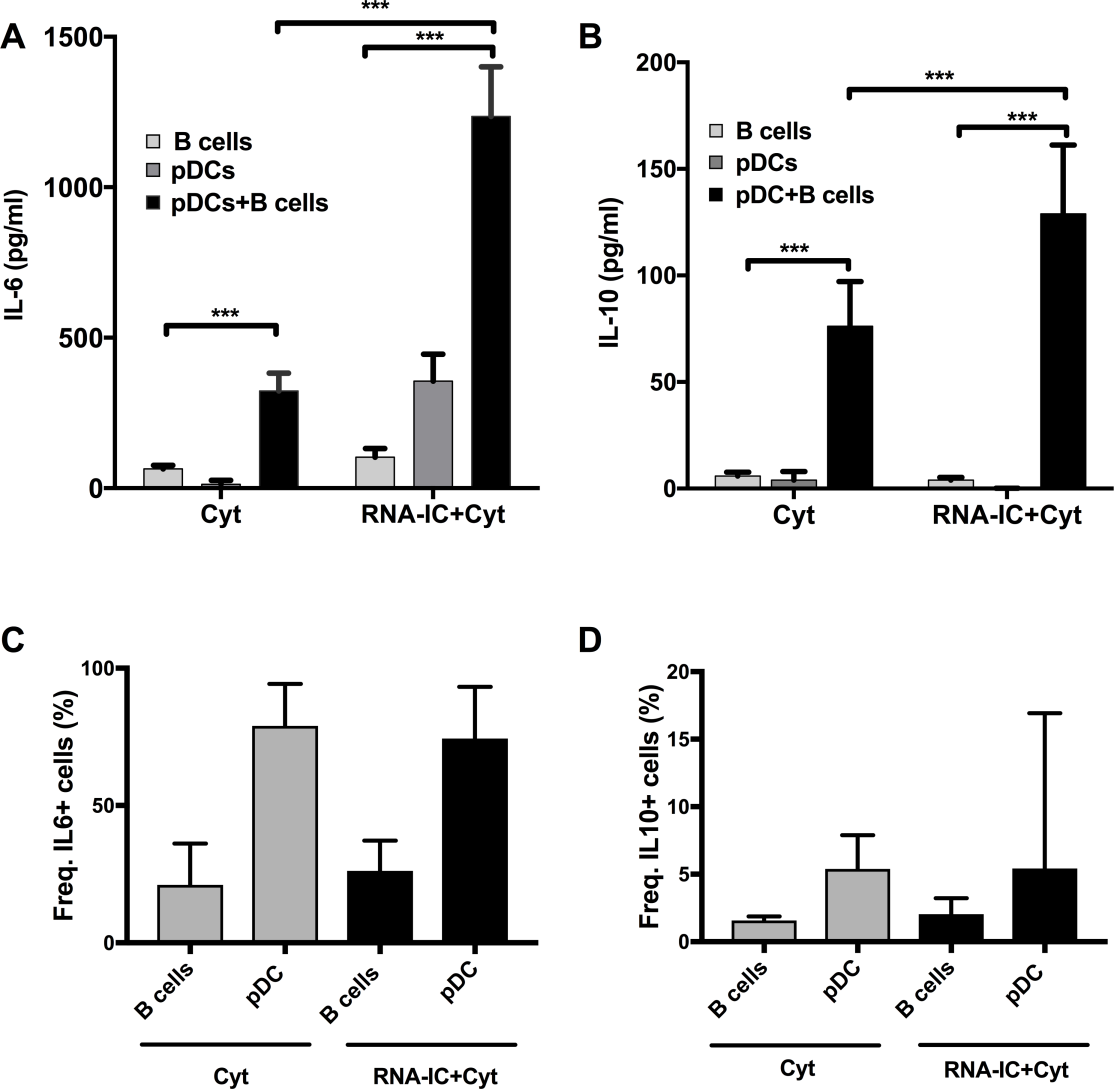
Increased IL-6 and IL-10 production by co-cultivated B cells and plasmacytoid dendritic cells. B cells and pDCs were isolated from healthy donor PBMCs. (A) The B cells were cultivated alone or (A, B) in co-cultures with pDCs for 6 days with IL-3/GM-CSF (Cyt), and in absence or presence of RNA-containing immune complexes (RNA-IC). Production of the cytokines (A) IL-6 and (B) IL-10 in the cell cultures were determined by immunoassays. (C, D) The frequency of (C) IL-6 or (D) IL-10 producing pDCs and B cells in the co-cultures was determines by flow cytometry. Mean values ± SEM based on 7–11 individual donors are shown in (A) and (B). The frequencies of IL-6 and IL-10 positive cells are based on (C) 3–6 and (D) 7–9 individual donors * = p<0.05, ** = p<0.01, *** = p<0.001. Statistical analyses were performed by Wilcoxon signed rank test.

The highest levels of IL-6 (Fig 4A), IL-10 (Fig 4B) and IFN-α (S2 Fig) were found in the co-cultures of B cells and pDCs, particularly when stimulated with RNA-IC. To determine the cellular source of IL-6 and IL-10 in the RNA-IC-stimulated co-cultures the B cells and pDCs were analyzed for intracellular IL-6 and IL-10 at day 6 by flow cytometry. We found that 26% (SD±11%) of the B cells and 74% (SD±19%) of the pDCs were positive for intracellular IL-6 (Fig 4C) whereas 2% (SD±1%) of the B cells and 5% (SD±11%) of the pDCs were positive for IL-10 (Fig 4D). We also noticed that the frequency of IL-6 and IL-10 producing cells at the time point was not affected by the presence of RNA-IC.

Neither BAFF or TGF-β were detected in any cell cultures (results not shown). To more thoroughly investigate whether low levels of BAFF undetectable by the immunoassay could contribute to the increase of the double negative B cells we added neutralizing antibodies to BAFF or control IgG into the cell cultures. No effect of the antibodies was detected on the increase of the double negative CD27^-^IgD^-^ B cells in the RNA-IC stimulated co-cultures at day 6 (S3 Fig).

Thus, activation of B cells and pDCs by RNA-IC containing immune complexes enhanced the expression of the co-stimulatory molecule CD86 and the production of cytokines IL-10, IL-6 and IFN-α all being important regulators of B cell functions.

### Increased frequency of CD24^hi^CD38^hi^ B cells in co-cultures with pDCs

Production of IL-10 is an important functional marker of regulatory B cells (Bregs) [25]. Since the IL-10 production was enhanced in the co-cultures of B cells and pDCs stimulated with RNA-IC we examined whether development of Bregs, characterized as CD24^hi^CD38^hi^ B cells [16], was supported under these conditions. We found an increased frequency of the CD24^hi^CD38^hi^ B cells in the co-cultures (12% ±5.9) compared to B cells cultured alone (7% ± 4, mean±SD, p< 0.001) whereas the RNA-IC did not have any additional stimulatory effect (results not shown).

Thus, our data are in line with previous studies showing that interactions between B cells and pDCs increased the IL-10 production and the frequency of the CD24^hi^CD38^hi^ B cells [13, 26].

### Gene expression profile of the double negative CD27^-^IgD^-^ and the switched memory CD27^+^IgD^-^ B cells

The relative proportion of both double negative CD27^-^IgD^-^ B cells and switched memory CD27^+^IgD^-^ B cells are increased in active SLE [1]. Particularly, the impact of the double negative CD27^-^IgD^-^ B cells still remains to be clarified. To reveal possible functional characteristics of the double negative CD27^-^IgD^-^ B cells we finally performed a mRNA expression profiling of the double negative CD27^-^IgD^-^ B cells and used the switched memory CD27^+^IgD^-^ B cells from the same individuals for comparison. The cells were isolated by flow cytometric cell sorting from co-cultures of B cells and pDCs stimulated with RNA-IC for four days (S4 Fig). The expression of 614 genes involved in immunological pathways was analyzed by nCounter expression array (S1 Table).

**Fig 5:**
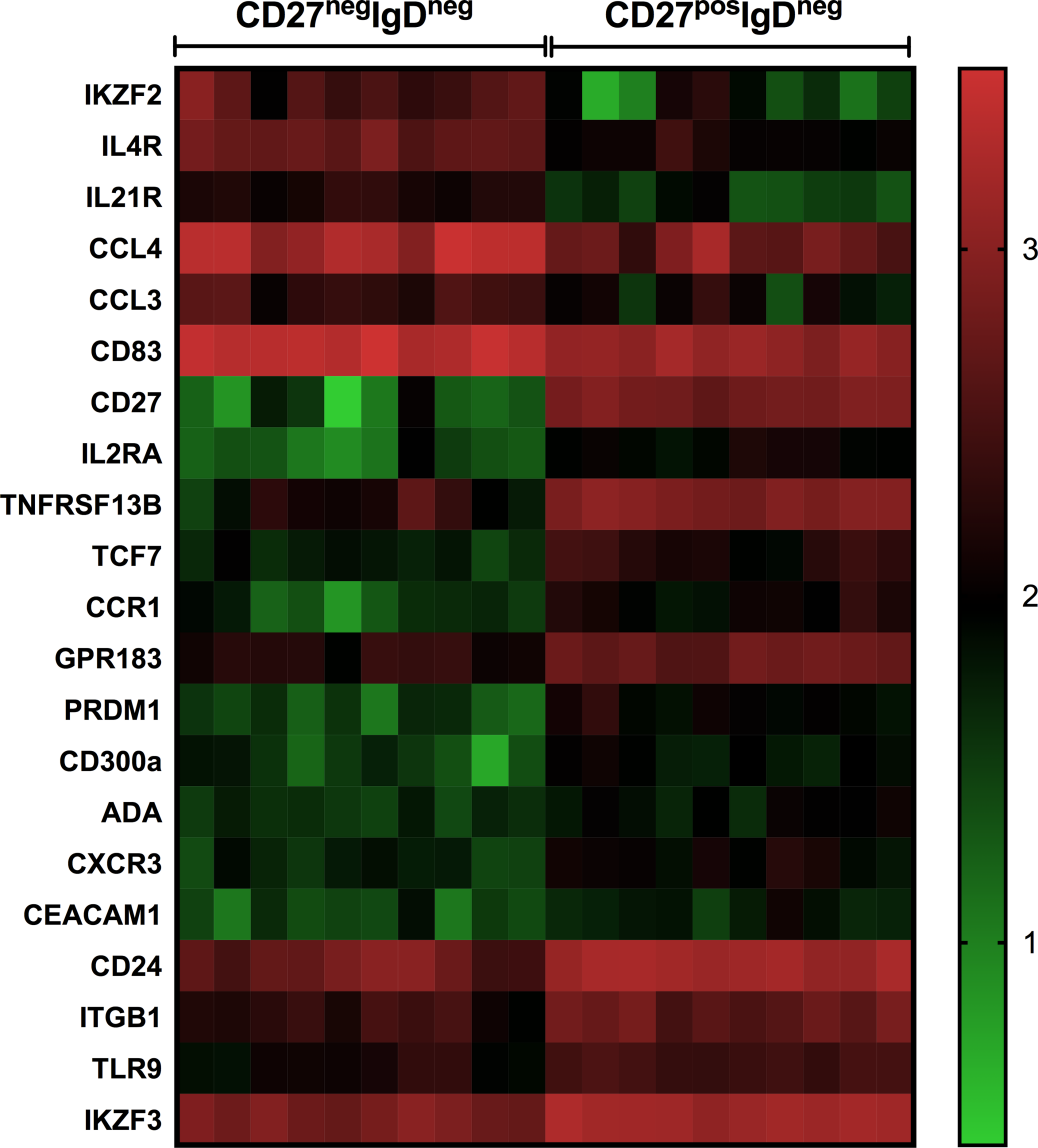
Twenty one genes differentially expressed by double negative CD27^-^IgD^-^ B cells and switched memory CD27^+^IgD^-^ B cells. Heat map of gene expression levels by double negative CD27^-^IgD^-^ B cells (CD27^neg^IgD^neg^) and switched memory B cells (CD27^pos^IgD^neg^) (genes with >2-fold difference, p≤0.001). Plasmacytoid dendritic cells and CD19+ B cells were isolated from peripheral blood of healthy individuals (n = 10) and co-cultivated in the presence of RNA-containing immune complexes (RNA-IC) for four days. The CD27^neg^IgD^neg^ and CD27^pos^IgD^neg^ B cell subsets were isolated by flow cytometric cell sorting after staining with antibodies to CD123, CD19, CD27 and IgD. The mRNA expression of totally 614 genes were analyzed by nCounter gene expression platform and nSolverAnalysis Software 3.0. Each column represents a sample and rows represent differentially expressed genes. Median differences between the groups were analyzed by using Wilcoxon signed rank test.

When comparing mRNA expression levels of the double negative CD27^-^IgD^-^ and switched memory CD27^+^IgD^-^ B cells, 21 genes showed differential expression (≥2-fold difference, p<0.001) (Fig 5). Six genes, *IL21R*, *IL4R*, *C-C Motif Chemokine Ligand* (*CCL)4*, *CCL3*, *CD83* and the *IKAROS Family Zinc Finger* (*IKZF)2* were expressed at higher levels by the double negative B cells (Fig 5, S2 Table). Among these gene transcripts, *IKZF2* displayed the largest difference in counts between the CD27^-^IgD^-^ B cells (median 367, range 97–983) and CD27^+^IgD^-^ B cells (median <50, range <50–190), followed by the *IL4R*, *IL21R* and *CCL4* that were expressed more than 4-fold higher levels in the double negative B cells. (Fig 5, S2 Table). The activation marker *CD83* and *CCL4* (MIP-1β*)* showed the highest expression among these six genes.

In addition, *Inducible T-Cell Costimulator Ligand* (*ICOSL)* and *Signaling Lymphocytic Activation Molecule Family Member 1* (*SLAMF1)* were expressed at 2-fold higher levels in the double negative B cells although the difference was slightly less significant (p<0.01) (S2 Table). Interestingly, IL-21R, IL-4R, CD83, ICOSL and SLAM molecules are involved in interactions between B cells and follicular T helper cells in germinal centers [27, 28].

Fifteen genes displayed higher expression in the isotype-switched CD27^+^IgD^-^ memory B cells (p<0.001) compared with the double negative B cells (Fig 5, S2 Table).

These genes included e.g the BAFF-receptor *Tumor Necrosis Factor Receptor Superfamily Member 13B* (*TNFRSF13B*/TACI) that was expressed more than six times higher level. Other genes that were highly expressed by the CD27^+^IgD^-^ B cells were *IKZF3 (IKAROS)*, *CD24* and the *chemotactic G-Protein Coupled Receptor 183 (GPR183)* (Fig 5, S2 Table). Also TLR9 was more strongly expressed by the switched memory B cells in comparison with the CD27^-^IgD^-^ B cells.

Several gene transcripts that were expressed by switched memory B cells had very low or no expression in the CD27^-^IgD^-^ B cells, e.g. *CD300a* and the *Positive Regulatory Domain Zinc Finger Protein 1 (PRDM1/BLIMP-1)*, both mediating inhibitory signals in B cells [29]. The double negative B cells also lacked expression of the chemokine receptor *CXCR3* (IP-10R) and the co-inhibitory receptor *CEACAM1* that were expressed at low levels by the CD27^+^IgD^-^ B cells.

In summary, several genes markedly expressed by the expanded double negative CD27^-^IgD^-^ B cells in presence of RNA-IC and pDCs are involved in activation and differentiation of B cells, as well as in interactions with T cells and other immune cells.

## Discussion

An intriguing observation in SLE is that the double negative CD27^-^IgD^-^ B cells can be increased to 30–40% of the peripheral blood B cells [7] and that this increase is connected to an active disease phenotype. However, the exact mechanism driving the development of such B cells has not been defined.

In the present study, we found a more than 5-fold increased frequency of double negative CD27^-^IgD^-^ B cells, from 7% among freshly isolated B cells to almost 40% of the total B cell population, in co-cultures with pDCs stimulated with RNA-IC. We also detected a significant increase of CD95 expressing B cells in the stimulated co-cultures. This is in analogy with previous studies in SLE showing that double negative CD27^-^IgD^-^ memory B cells with enhanced CD95 expression had an activated profile [7, 24, 30]. The mechanism driving the development of CD27^-^IgD^-^ B cells has been suggested to depend on extra follicular BAFF production by conventional DCs triggered by IFN-α, which can mediate differentiation and survival of autoreactive B cells [31]. However, we could not detect any BAFF in the cell cultures and neutralizing antibodies to BAFF did not block the increase of the double negative CD27^-^IgD^-^ B cells in the RNA-IC stimulated co-cultures of B cells and pDCs. Thus, the expansion of the double negative B cells in our study does not appear to be driven by BAFF. Furthermore, neither RNA-IC alone nor IFN-α (not shown) could efficiently facilitate the increase of double negative CD27^-^IgD^-^ B cells whereas interaction with activated pDCs together with RNA-IC markedly increased their expansion. Accordingly, expression of CD86, and production of IL-6 and IFN-α were increased upon cultivation with activated pDCs, and such co-stimulation can modulate differentiation and survival of autoreactive B cells and affect their communication with other immune cells.

Several studies have shown that stimulation of pDCs and B cells with CpG DNA TLR agonists and/or CD40 ligation can trigger development of IL-10 producing Bregs [13, 26, 32]. Here we asked whether the physiological TLR7 stimulators i.e RNA-IC and pDCs could induce a pronounced regulatory B cell profile. The enhanced CD86 expression by B cells upon interaction with pDCs is also in favor of the achieved regulatory profile. It has been stated that the number of Bregs is increased in SLE but that their functionality is poor [13, 16]. The mean level of IL-10 in the RNA-IC-stimulated co-cultures was 130 pg/ml whilst it was 4 times higher when CpG ODN2216 was used as stimuli (not shown). One explanation to the moderate IL-10 production by B cells in the RNA-IC stimulated co-cultures could be the high levels of IFN-α in these cultures. Therefore, our findings could mimic the state in several autoimmune diseases with continuously IC-activated pDCs and an increased number of regulatory B cells with impaired capacity to produce IL-10 and suppress extensive immune activation.

To our knowledge, a comprehensive mRNA expression profile of CD27^-^IgD^-^ B cells has not been performed previously. To examine the characteristics of the CD27^-^IgD^-^ B cells enriched in the RNA-IC stimulated co-cultures with pDCs we performed gene expression profiling of sorted CD27^-^IgD^-^ B cells and analyzed switched memory CD27^+^IgD^-^ B cells for comparison. We found that expression levels of 21 gene transcripts differed between the two B cells subsets (p<0.001). Six genes were expressed ≥ 2-fold higher level in the CD27^-^IgD^-^ B cells, namely *IL21R*, *IL4R*, *IKZF2*, *CCL3*, *CCL4* and *CD83*. Interestingly, IL21R and IL4R as well as the co-stimulatory molecules CD83 and CD86 are involved in the signaling between B cells and follicular T helper cells (T_FH_) cells [5, 27]. Especially, T_FH_ cells are effective producers of IL-21 and ICOS that provide essential help for development of extrafollicular plasma cells as well as generation of high-affinity memory B cells and long lived plasma cells in the germinal centers. In this context, it was interesting that both *ICOSL* and *SLAMF1* were expressed at higher levels compared with switched memory B cells, although the difference did not reach the same significance level. Furthermore, we isolated CD27^-^IgD^-^ and CD27^+^IgD^-^ B cells from 3 patients with SLE and found the same trend for expression of *CD83*, *ICOSL*, *SLAMF1*, and *IL-21R* (results not shown), suggesting that our in vitro generated CD27^-^IgD^-^ and CD27^+^IgD^-^ B cells mimic the B cells seen in SLE.

Strong expression of IL-21R, ICOSL, IL4R and SLAMF1 by CD27^-^IgD^-^ B cells in combination with IL-6 and IFN-α production could potentially enhance the responsiveness of double negative B cells to co-stimulatory signals from e.g. T_FH_ cells and trigger their activation and survival. Especially, when keeping in mind that patients with SLE have increased serum levels of IL-21 as well as IL-6 and IFN-α [3, 33].

Furthermore, the gene expression profiling of CD27^-^IgD^-^ memory B cells and CD27^+^IgD^-^ memory B cells revealed that the double negative B cells had very low or absent expression of several inhibitory molecules, for example the genes encoding the transcriptional repressor PRDM1/BLIMP-1 and CD300a [29, 34]. Taken together the gene expression profile of the double negative CD27^-^IgD^-^ B cells indicates that they possess a phenotype that facilitates their recruitment to GC and interactions with other activated immune cells.

In summary, our results suggest that in an environment with RNA-containing IC the interaction between B cells and pDCs results in a remarkable increase of B cells with a phenotype typical for autoreactive B lymphocytes seen in patients with SLE. A milieu with pDCs and ICs that stimulate B cells will at the same time support the development of Bregs with impaired IL-10 producing capacity. Possible consequences could be prolonged survival and activation of B cell functions, such as antigen presentation, production of cytokines and ligands leading to delivery of co-stimulatory signals to surrounding immune cells, including those prone to autoimmune reactivity. We conclude that all these events could promote the autoimmune disease process present in SLE.

## Supporting information

S1 Fig Gating strategy for flow cytometric analysis of co-cultured B cells and plasmacytoid dendritic cells. The plasmacytoid dendritic cells (pDCs) and B cells from healthy blood donors were cultured in co-cultures or alone for 6 days in presence of IL-3/GM-CSF and in absence or presence of RNA-containing immune complexes (RNA-IC). The cells were stained with monoclonal antibodies to CD19, CD123 and the LIVE/DEAD near-IR dead cell stain. The cells were first gated as singlets, live cells and as CD19^+^ B cells or CD123^+^ pDCs.
(TIFF)

S2 Fig Interferon-α production in co-cultures of B cells and plasmacytoid dendritic cells. Production of IFN-α by plasmacytoid dendritic cells (pDCs), alone or in co-cultures with B cells stimulated for 6 days with IL-3/GM-CSF (Cyt) and in absence or presence of RNA containing immune complexes (RNA-IC). The IFN-α level was measured in the culture supernatants by an immunoassay. Mean values ± SEM based on 7–11 individual donors are shown. Statistical analyses were performed by Wilcoxon signed rank test. B cells alone did not produce any detectable levels (≥ 2 U/ml) of IFN-α.
(TIFF)

S3 Fig Neutralizing antibodies to BAFF do not affect the increase of double negative CD27^-^IgD^-^ B cells in co-cultures with plasmacytoid dendritic cells. Plasmacytoid dendritic cells (pDCs) and CD19+ B cells isolated from healthy blood donors were cultured in co-cultures in presence of IL-3/GM-CSF and RNA-containing immune complexes. Polyclonal goat anti-BAFF antibodies or normal goat IgG were added into the cell cultures (20 μg/ml) at the beginning of the culturing period. At day six the cells were stained with monoclonal antibodies to CD19, IgD, CD27, CD123 and the LIVE/DEAD near-IR dead cell stain, and analyzed by flow cytometry. The cells were first gated as singlets, live cells and as CD19^+^ B cells. Frequency of the double negative B cells (mean (%)±SEM) from three individual donors is shown.
(TIFF)

S4 Fig Expansion of the double negative CD27^-^IgD^-^ B cells in vitro over time. The frequency of double negative CD27^-^IgD^-^ B cells in the total CD19^+^ B cell population was determined by flow cytometry after staining with monoclonal antibodies to CD19, CD27 and IgD at day 0 or after 1, 3, 4 or 6 days of co-culture with pDCs.
(TIFF)

S1 Table nCounter human immunology V2 panel gene list and the additional 20 genes included in the Custom CodeSet (nCounter, NanoString). (PDF)

S2 Table Median values (n = 10) of gene counts from nCounter expression array (NanoString) of CD27negIgDneg and CD27posIgDneg B cells. (PDF)
