# Supplementary figures and images for: Plasmacytoid dendritic cells and RNA-containing immune complexes drive expansion of peripheral B cell subsets with an SLE-like phenotype
Source: PLoS One. 2017 Aug 28;12(8):e0183946. doi: 10.1371/journal.pone.0183946 (PMC5573130; doi:10.1371/journal.pone.0183946)

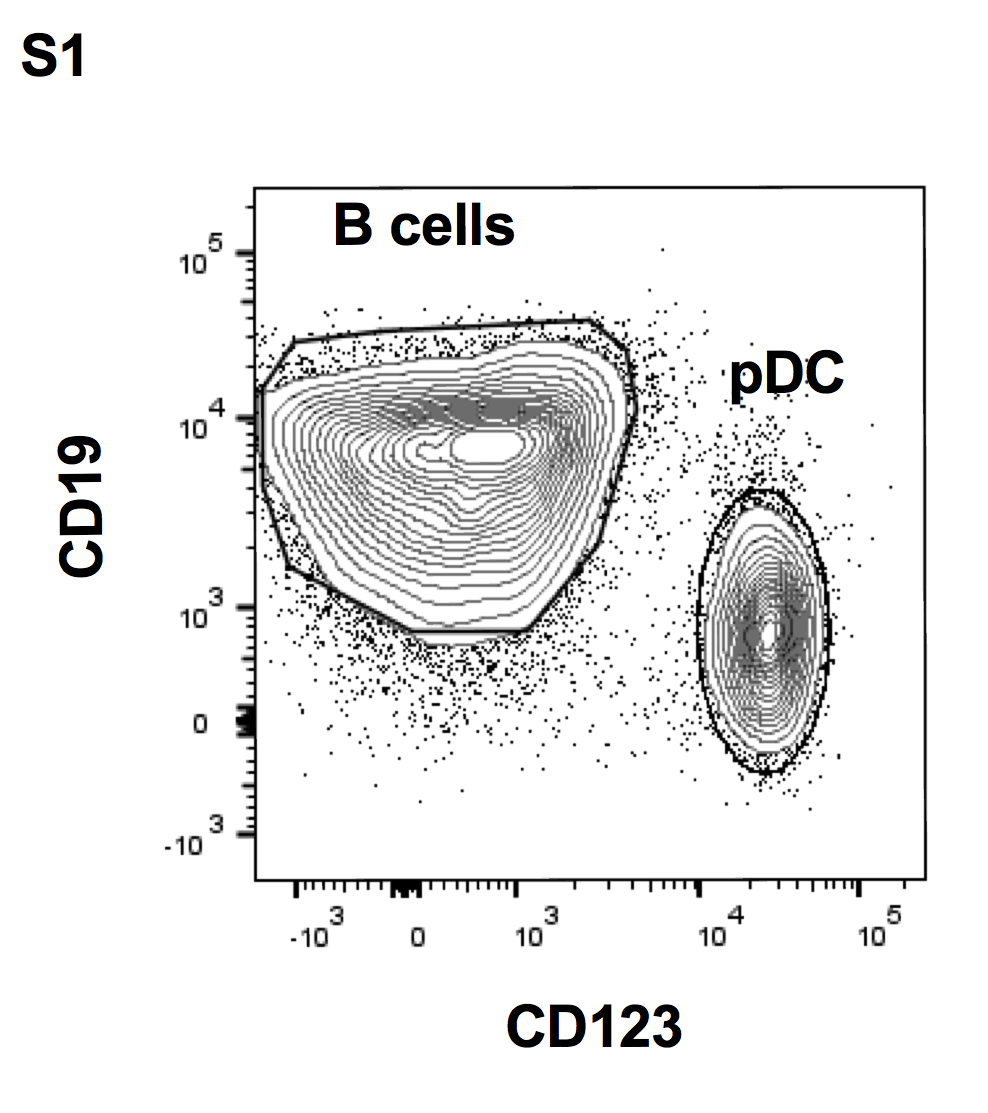

Supplement: S1 Fig — The plasmacytoid dendritic cells (pDCs) and B cells from healthy blood donors were cultured in co-cultures or alone for 6 days in presence of IL-3/GM-CSF and in absence or presence of RNA-containing immune complexes (RNA-IC). The cells were stained with monoclonal antibodies to CD19, CD123 and the LIVE/DEAD near-IR dead cell stain. The cells were first gated as singlets, live cells and as CD19+ B cells or CD123+ pDCs. (TIFF) [file pone.0183946.s001.tiff]

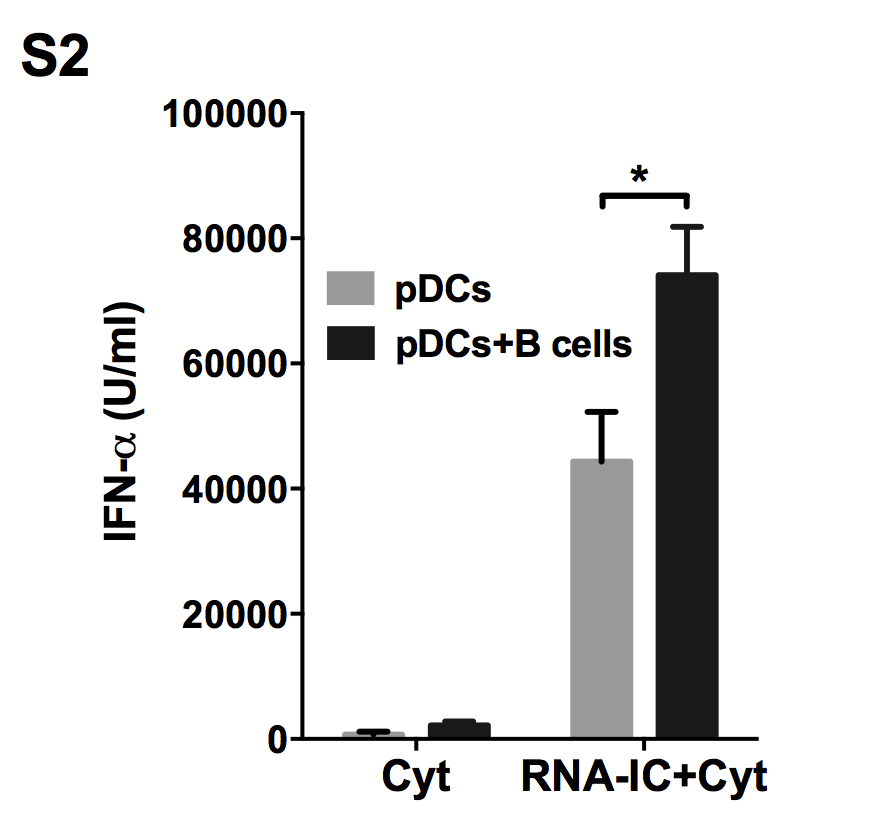

Supplement: S2 Fig — Production of IFN-α by plasmacytoid dendritic cells (pDCs), alone or in co-cultures with B cells stimulated for 6 days with IL-3/GM-CSF (Cyt) and in absence or presence of RNA containing immune complexes (RNA-IC). The IFN-α level was measured in the culture supernatants by an immunoassay. Mean values ± SEM based on 7–11 individual donors are shown. Statistical analyses were performed by Wilcoxon signed rank test. B cells alone did not produce any detectable levels (≥ 2 U/ml) of IFN-α. (TIFF) [file pone.0183946.s002.tiff]

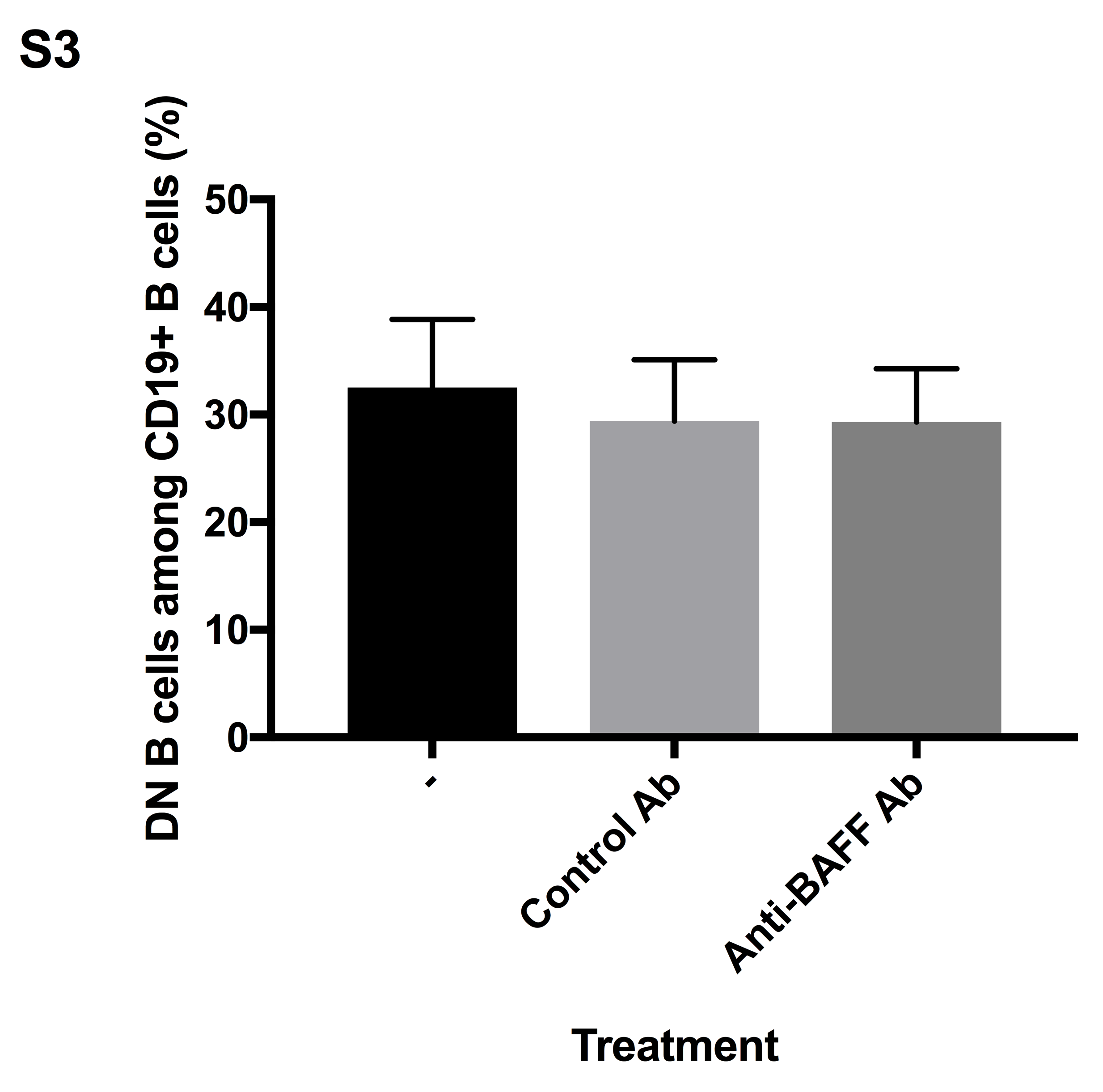

Supplement: S3 Fig — Plasmacytoid dendritic cells (pDCs) and CD19+ B cells isolated from healthy blood donors were cultured in co-cultures in presence of IL-3/GM-CSF and RNA-containing immune complexes. Polyclonal goat anti-BAFF antibodies or normal goat IgG were added into the cell cultures (20 μg/ml) at the beginning of the culturing period. At day six the cells were stained with monoclonal antibodies to CD19, IgD, CD27, CD123 and the LIVE/DEAD near-IR dead cell stain, and analyzed by flow cytometry. The cells were first gated as singlets, live cells and as CD19+ B cells. Frequency of the double negative B cells (mean (%)±SEM) from three individual donors is shown. (TIFF) [file pone.0183946.s003.tiff]

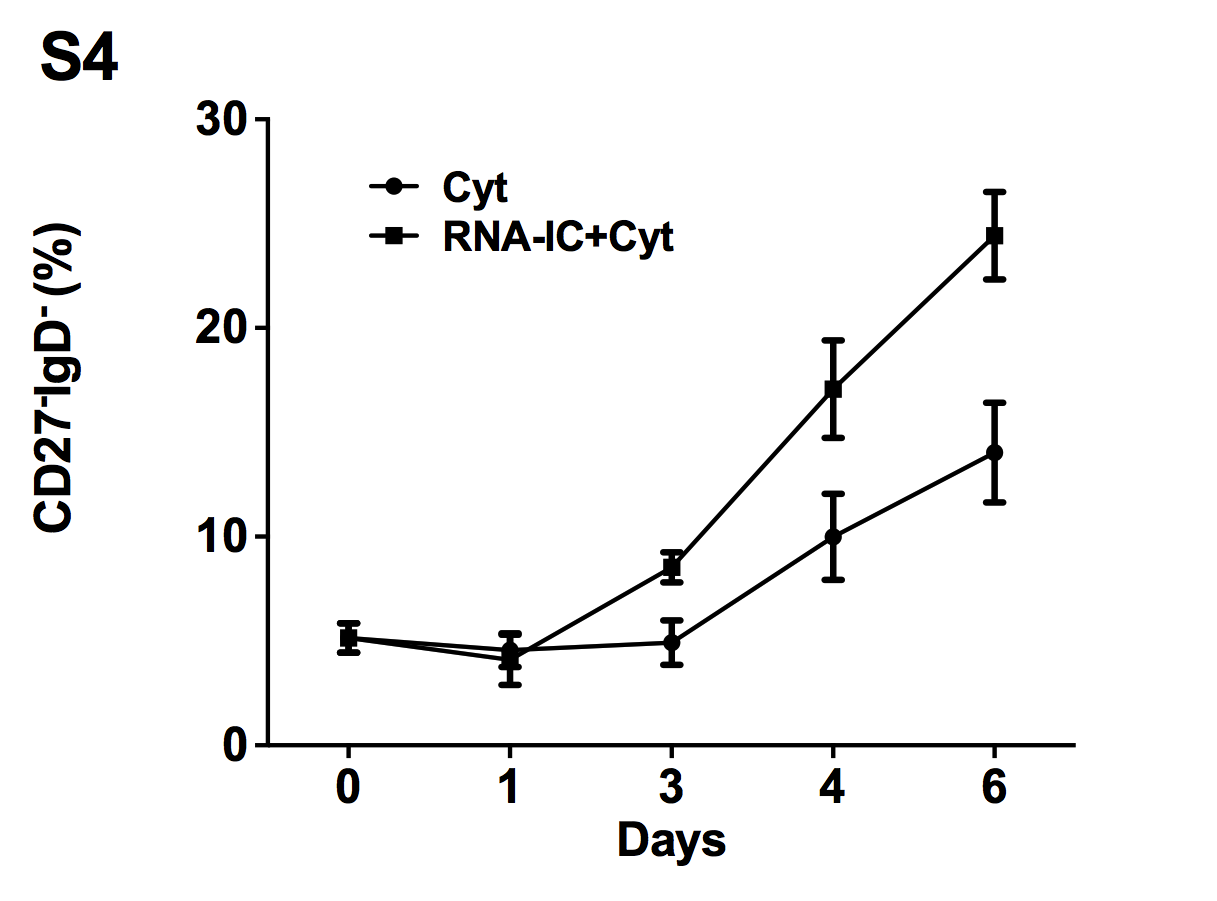

Supplement: S4 Fig — The frequency of double negative CD27-IgD- B cells in the total CD19+ B cell population was determined by flow cytometry after staining with monoclonal antibodies to CD19, CD27 and IgD at day 0 or after 1, 3, 4 or 6 days of co-culture with pDCs. (TIFF) [file pone.0183946.s004.tiff]
