# Supplementary material for: Plasmacytoid dendritic cells and RNA-containing immune complexes drive expansion of peripheral B cell subsets with an SLE-like phenotype
Source: PLoS One. 2017 Aug 28;12(8):e0183946. doi: 10.1371/journal.pone.0183946 (PMC5573130; doi:10.1371/journal.pone.0183946)
Supplement: S1 Table — (PDF) [file pone.0183946.s005.pdf]

## Supplementary Table S1

**nCounter Human Immunology V2 Panel Gene List**
**and the 20 additional genes included in the Custom CodeSet (nCounter, NanoString)**

| Official Symbol | Accession |
|---|---|
| ABCB1 | NM_000927.3 |
| ABL1 | NM_005157.3 |
| ADA | NM_000022.2 |
| AHR | NM_001621.3 |
| AICDA | NM_020661.1 |
| AIRE | NM_000383.2 |
| APP | NM_000484.3 |
| ARG1 | NM_000045.2 |
| ARG2 | NM_001172.3 |
| ARHGDIB | NM_001175.4 |
| ATG10 | NM_001131028.1 |
| ATG12 | NM_004707.2 |
| ATG16L1 | NM_198890.2 |
| ATG5 | NM_004849.2 |
| ATG7 | NM_001136031.2 |
| ATM | NM_000051.3 |
| B2M | NM_004048.2 |
| B3GAT1 | NM_018644.3 |
| BATF | NM_006399.3 |
| BATF3 | NM_018664.2 |
| BAX | NM_138761.3 |
| BCAP31 | NM_005745.7 |
| BCL10 | NM_003921.2 |
| BCL2 | NM_000657.2 |
| BCL2L11 | NM_138621.4 |
| BCL3 | NM_005178.2 |
| BCL6 | NM_001706.2 |
| BID | NM_001196.2 |
| BLNK | NM_013314.2 |
| BST1 | NM_004334.2 |
| BST2 | NM_004335.2 |
| BTK | NM_000061.1 |
| BTLA | NM_181780.2 |
| C14orf166 | NM_016039.2 |
| C1QA | NM_015991.2 |
| C1QB | NM_000491.3 |
| C1QBP | NM_001212.3 |
| C1R | NM_001733.4 |
| C1S | NM_001734.2 |
| C2 | NM_000063.3 |
| C3 | NM_000064.2 |

| | |
|---|---|
| C4A/B | NM_007293.2 |
| C4BPA | NM_000715.3 |
| C5 | NM_001735.2 |
| C6 | NM_000065.2 |
| C7 | NM_000587.2 |
| C8A | NM_000562.2 |
| C8B | NM_000066.2 |
| C8G | NM_000606.2 |
| C9 | NM_001737.3 |
| CAMP | NM_004345.3 |
| CARD9 | NM_052813.4 |
| CASP1 | NM_001223.3 |
| CASP10 | NM_032977.3 |
| CASP2 | NM_032982.2 |
| CASP3 | NM_032991.2 |
| CASP8 | NM_001228.4 |
| CCBP2 | NM_001296.3 |
| CCL11 | NM_002986.2 |
| CCL13 | NM_005408.2 |
| CCL15 | NM_032965.3 |
| CCL16 | NM_004590.2 |
| CCL18 | NM_002988.2 |
| CCL19 | NM_006274.2 |
| CCL2 | NM_002982.3 |
| CCL20 | NM_004591.1 |
| CCL22 | NM_002990.3 |
| CCL23 | NM_145898.1 |
| CCL24 | NM_002991.2 |
| CCL26 | NM_006072.4 |
| CCL3 | NM_002983.2 |
| CCL4 | NM_002984.2 |
| CCL5 | NM_002985.2 |
| CCL7 | NM_006273.2 |
| CCL8 | NM_005623.2 |
| CCND3 | NM_001760.2 |
| CCR1 | NM_001295.2 |
| CCR10 | NM_016602.2 |
| CCR2 | NM_001123041.2 |
| CCR5 | NM_000579.1 |
| CCR6 | NM_031409.2 |
| CCR7 | NM_001838.2 |
| CCR8 | NM_005201.2 |
| CCRL1 | NM_016557.2 |
| CCRL2 | NM_003965.4 |
| CD14 | NM_000591.2 |
| CD160 | NM_007053.2 |

| | |
|---|---|
| CD163 | NM_004244.4 |
| CD164 | NM_006016.4 |
| CD19 | NM_001770.4 |
| CD1A | NM_001763.2 |
| CD1D | NM_001766.3 |
| CD2 | NM_001767.3 |
| CD209 | NM_021155.2 |
| CD22 | NM_001771.2 |
| CD24 | NM_013230.2 |
| CD244 | NM_016382.2 |
| CD247 | NM_198053.1 |
| CD27 | NM_001242.4 |
| CD274 | NM_014143.3 |
| CD276 | NM_001024736.1 |
| CD28 | NM_001243078.1 |
| CD34 | NM_001025109.1 |
| CD36 | NM_001001548.2 |
| CD3D | NM_000732.4 |
| CD3E | NM_000733.2 |
| CD3EAP | NM_012099.1 |
| CD4 | NM_000616.4 |
| CD40 | NM_001250.4 |
| CD40LG | NM_000074.2 |
| CD44 | NM_001001392.1 |
| CD46 | NM_172350.1 |
| CD48 | NM_001778.2 |
| CD5 | NM_014207.2 |
| CD53 | NM_001040033.1 |
| CD55 | NM_000574.3 |
| CD58 | NM_001779.2 |
| CD59 | NM_000611.4 |
| CD6 | NM_006725.3 |
| CD7 | NM_006137.6 |
| CD70 | NM_001252.2 |
| CD74 | NM_001025159.1 |
| CD79A | NM_001783.3 |
| CD79B | NM_021602.2 |
| CD80 | NM_005191.3 |
| CD81 | NM_004356.3 |
| CD82 | NM_002231.3 |
| CD83 | NM_004233.3 |
| CD86 | NM_175862.3 |
| CD8A | NM_001768.5 |
| CD8B | NM_004931.3 |
| CD9 | NM_001769.2 |
| CD96 | NM_005816.4 |

| | |
|---|---|
| CD97 | NM_078481.2 |
| CD99 | NM_002414.3 |
| CDH5 | NM_001795.3 |
| CDKN1A | NM_000389.2 |
| CEACAM1 | NM_001712.3 |
| CEACAM6 | NM_002483.4 |
| CEACAM8 | NM_001816.3 |
| CEBPB | NM_005194.2 |
| CFB | NM_001710.5 |
| CFD | NM_001928.2 |
| CFH | NM_001014975.2 |
| CFI | NM_000204.3 |
| CFP | NM_002621.2 |
| CHUK | NM_001278.3 |
| CIITA | NM_000246.3 |
| CISH | NM_145071.2 |
| CLEC4A | NM_194448.2 |
| CLEC4E | NM_014358.2 |
| CLEC5A | NM_013252.2 |
| CLEC6A | NM_001007033.1 |
| CLEC7A | NM_197954.2 |
| CLU | NM_001831.2 |
| CMKLR1 | NM_004072.1 |
| CR1 | NM_000651.4 |
| CR2 | NM_001006658.1 |
| CRADD | NM_003805.3 |
| CSF1 | NM_000757.4 |
| CSF1R | NM_005211.2 |
| CSF2 | NM_000758.2 |
| CSF2RB | NM_000395.2 |
| CSF3R | NM_156038.2 |
| CTLA4_all (comm | NM_005214.3 |
| CTLA4-TM (memb | NM_005214.3 |
| sCTLA4 (soluble fc | NM_001037631.1 |
| CTNNB1 | NM_001098210.1 |
| CTSC | NM_001814.4 |
| CTSG | NM_001911.2 |
| CTSS | NM_004079.3 |
| CUL9 | NM_015089.2 |
| CX3CL1 | NM_002996.3 |
| CX3CR1 | NM_001337.3 |
| CXCL1 | NM_001511.1 |
| CXCL10 | NM_001565.1 |
| CXCL11 | NM_005409.4 |
| CXCL12 | NM_000609.5 |
| CXCL13 | NM_006419.2 |

| | |
|---|---|
| CXCL2 | NM_002089.3 |
| CXCL9 | NM_002416.1 |
| CXCR1 | NM_000634.2 |
| CXCR2 | NM_001557.2 |
| CXCR3 | NM_001504.1 |
| CXCR4 | NM_003467.2 |
| CXCR6 | NM_006564.1 |
| CYBB | NM_000397.3 |
| DEFB1 | NM_005218.3 |
| DEFB103A | NM_001081551.2 |
| DEFB103B | NM_018661.3 |
| DEFB4A | NM_004942.2 |
| DPP4 | NM_001935.3 |
| DUSP4 | NM_057158.2 |
| EBI3 | NM_005755.2 |
| EDNRB | NM_003991.2 |
| EGR1 | NM_001964.2 |
| EGR2 | NM_000399.3 |
| ENTPD1 | NM_001098175.1 |
| EOMES | NM_005442.2 |
| ETS1 | NM_005238.3 |
| FADD | NM_003824.2 |
| FAS | NM_000043.3 |
| FCAR | NM_133280.1 |
| FCER1A | NM_002001.2 |
| FCER1G | NM_004106.1 |
| FCGR1A/B | NM_000566.3 |
| FCGR2A | NM_021642.3 |
| FCGR2A/C | NM_201563.4 |
| FCGR2B | NM_001002273.1 |
| FCGR3A/B | NM_000570.4 |
| FCGRT | NM_004107.4 |
| FKBP5 | NM_001145775.1 |
| FN1 | NM_212482.1 |
| FOXP3 | NM_014009.3 |
| FYN | NM_002037.3 |
| GATA3 | NM_001002295.1 |
| GBP1 | NM_002053.1 |
| GBP5 | NM_052942.3 |
| GFI1 | NM_005263.2 |
| GNLY | NM_006433.2 |
| GP1BB | NM_000407.4 |
| GPI | NM_000175.2 |
| GPR183 | NM_004951.3 |
| GZMA | NM_006144.2 |
| GZMB | NM_004131.3 |

| | |
|---|---|
| GZMK | NM_002104.2 |
| HAMP | NM_021175.2 |
| HAVCR2 | NM_032782.3 |
| HFE | NM_139011.2 |
| HLA-A | NM_002116.5 |
| HLA-B | NM_005514.6 |
| HLA-C | NM_002117.4 |
| HLA-DMA | NM_006120.3 |
| HLA-DMB | NM_002118.3 |
| HLA-DOB | NM_002120.3 |
| HLA-DPA1 | NM_033554.2 |
| HLA-DPB1 | NM_002121.4 |
| HLA-DQA1 | NM_002122.3 |
| HLA-DQB1 | NM_002123.3 |
| HLA-DRA | NM_019111.3 |
| HLA-DRB1 | NM_002124.2 |
| HLA-DRB3 | NM_022555.3 |
| HRAS | NM_005343.2 |
| ICAM1 | NM_000201.2 |
| ICAM2 | NM_000873.3 |
| ICAM3 | NM_002162.3 |
| ICAM4 | NM_001039132.1 |
| ICAM5 | NM_003259.3 |
| ICOS | NM_012092.2 |
| ICOSLG | NM_015259.4 |
| IDO1 | NM_002164.3 |
| IFI16 | NM_005531.1 |
| IFI35 | NM_005533.3 |
| IFIH1 | NM_022168.2 |
| IFIT2 | NM_001547.4 |
| IFITM1 | NM_003641.3 |
| IFNA1/13 | NM_024013.1 |
| IFNA2 | NM_000605.3 |
| IFNAR1 | NM_000629.2 |
| IFNAR2 | NM_000874.3 |
| IFNB1 | NM_002176.2 |
| IFNG | NM_000619.2 |
| IFNGR1 | NM_000416.1 |
| IGF2R | NM_000876.1 |
| IKBKAP | NM_003640.3 |
| IKBKB | NM_001556.1 |
| IKBKE | NM_014002.2 |
| IKBKG | NM_003639.2 |
| IKZF1 | NM_006060.3 |
| IKZF2 | NM_016260.2 |
| IKZF3 | NM_183232.2 |

| | |
|---|---|
| IL10 | NM_000572.2 |
| IL10RA | NM_001558.2 |
| IL11RA | NM_147162.1 |
| IL12A | NM_000882.2 |
| IL12B | NM_002187.2 |
| IL12RB1 | NM_005535.1 |
| IL13 | NM_002188.2 |
| IL13RA1 | NM_001560.2 |
| IL15 | NM_172174.1 |
| IL16 | NM_004513.4 |
| IL17A | NM_002190.2 |
| IL17B | NM_014443.2 |
| IL17F | NM_052872.3 |
| IL18 | NM_001562.2 |
| IL18R1 | NM_003855.2 |
| IL18RAP | NM_003853.2 |
| IL19 | NM_013371.3 |
| IL1A | NM_000575.3 |
| IL1B | NM_000576.2 |
| IL1R1 | NM_000877.2 |
| IL1R2 | NM_173343.1 |
| IL1RAP | NM_002182.2 |
| IL1RL1 | NM_016232.4 |
| IL1RL2 | NM_003854.2 |
| IL1RN | NM_000577.3 |
| IL2 | NM_000586.2 |
| IL20 | NM_018724.3 |
| IL21 | NM_021803.2 |
| IL21R | NM_021798.2 |
| IL22 | NM_020525.4 |
| IL22RA2 | NM_181310.1 |
| IL23A | NM_016584.2 |
| IL23R | NM_144701.2 |
| IL26 | NM_018402.1 |
| IL27 | NM_145659.3 |
| IL28A | NM_172138.1 |
| IL28A/B | NM_172139.2 |
| IL29 | NM_172140.1 |
| IL2RA | NM_000417.1 |
| IL2RB | NM_000878.2 |
| IL2RG | NM_000206.1 |
| IL3 | NM_000588.3 |
| IL32 | NM_001012633.1 |
| IL4 | NM_000589.2 |
| IL4R | NM_000418.2 |
| IL5 | NM_000879.2 |

| | |
|---|---|
| IL6 | NM_000600.1 |
| IL6R | NM_000565.2 |
| IL6ST | NM_002184.2 |
| IL7 | NM_000880.2 |
| IL7R | NM_002185.2 |
| IL8 | NM_000584.2 |
| IL9 | NM_000590.1 |
| ILF3 | NM_001137673.1 |
| IRAK1 | NM_001569.3 |
| IRAK2 | NM_001570.3 |
| IRAK3 | NM_007199.1 |
| IRAK4 | NM_016123.1 |
| IRF1 | NM_002198.1 |
| IRF3 | NM_001571.5 |
| IRF4 | NM_002460.1 |
| IRF5 | NM_002200.3 |
| IRF7 | NM_001572.3 |
| IRF8 | NM_002163.2 |
| IRGM | NM_001145805.1 |
| ITGA2B | NM_000419.3 |
| ITGA4 | NM_000885.4 |
| ITGA5 | NM_002205.2 |
| ITGA6 | NM_000210.1 |
| ITGAE | NM_002208.4 |
| ITGAL | NM_002209.2 |
| ITGAM | NM_000632.3 |
| ITGAX | NM_000887.3 |
| ITGB1 | NM_033666.2 |
| ITGB2 | NM_000211.2 |
| ITLN1 | NM_017625.2 |
| ITLN2 | NM_080878.2 |
| JAK1 | NM_002227.1 |
| JAK2 | NM_004972.2 |
| JAK3 | NM_000215.2 |
| KCNJ2 | NM_000891.2 |
| KIR_Activating_Su | NM_001083539.1 |
| KIR_Activating_Su | NM_014512.1 |
| KIR_Inhibiting_Su | NM_014218.2 |
| KIR_Inhibiting_Su | NM_014511.3 |
| KIR3DL1 | NM_013289.2 |
| KIR3DL2 | NM_006737.2 |
| KIR3DL3 | NM_153443.3 |
| KIT | NM_000222.2 |
| KLRAP1 | NR_028045.1 |
| KLRB1 | NM_002258.2 |
| KLRC1 | NM_002259.3 |

| | |
|---|---|
| KLRC2 | NM_002260.3 |
| KLRC3 | NM_007333.2 |
| KLRC4 | NM_013431.2 |
| KLRD1 | NM_002262.3 |
| KLRF1 | NM_016523.1 |
| KLRF2 | NM_001190765.1 |
| KLRG1 | NM_005810.3 |
| KLRG2 | NM_198508.2 |
| KLRK1 | NM_007360.1 |
| LAG3 | NM_002286.5 |
| LAIR1 | NM_002287.3 |
| LAMP3 | NM_014398.3 |
| LCK | NM_005356.2 |
| LCP2 | NM_005565.3 |
| LEF1 | NM_016269.3 |
| LGALS3 | NM_001177388.1 |
| LIF | NM_002309.3 |
| LILRA1 | NM_006863.1 |
| LILRA2 | NM_006866.2 |
| LILRA3 | NM_006865.3 |
| LILRA4 | NM_012276.3 |
| LILRA5 | NM_181879.2 |
| LILRA6 | NM_024318.2 |
| LILRB1 | NM_001081637.1 |
| LILRB2 | NM_005874.1 |
| LILRB3 | NM_006864.2 |
| LILRB4 | NM_001081438.1 |
| LILRB5 | NM_001081442.1 |
| LITAF | NM_004862.3 |
| LTA | NM_000595.2 |
| LTB4R | NM_181657.3 |
| LTB4R2 | NM_019839.4 |
| LTBR | NM_002342.1 |
| LTF | NM_002343.2 |
| LY96 | NM_015364.2 |
| MAF | NM_005360.4 |
| MALT1 | NM_006785.2 |
| MAP4K1 | NM_007181.3 |
| MAP4K2 | NM_004579.2 |
| MAP4K4 | NM_004834.3 |
| MAPK1 | NM_138957.2 |
| MAPK11 | NM_002751.5 |
| MAPK14 | NM_001315.1 |
| MAPKAPK2 | NM_004759.3 |
| MARCO | NM_006770.3 |
| MASP1 | NM_139125.3 |

| | |
|---|---|
| MASP2 | NM_139208.1 |
| MBL2 | NM_000242.2 |
| MBP | NM_002385.2 |
| MCL1 | NM_021960.3 |
| MIF | NM_002415.1 |
| MME | NM_000902.2 |
| MR1 | NM_001531.2 |
| MRC1 | NM_002438.2 |
| MS4A1 | NM_152866.2 |
| MSR1 | NM_002445.3 |
| MUC1 | NM_001018017.1 |
| MX1 | NM_002462.2 |
| MYD88 | NM_002468.3 |
| NCAM1 | NM_000615.5 |
| NCF4 | NM_000631.4 |
| NCR1 | NM_004829.5 |
| NFATC1 | NM_172389.1 |
| NFATC2 | NM_012340.3 |
| NFATC3 | NM_004555.2 |
| NFIL3 | NM_005384.2 |
| NFKB1 | NM_003998.2 |
| NFKB2 | NM_002502.2 |
| NFKBIA | NM_020529.1 |
| NFKBIZ | NM_001005474.1 |
| NLRP3 | NM_001079821.2 |
| NOD1 | NM_006092.1 |
| NOD2 | NM_022162.1 |
| NOS2 | NM_000625.4 |
| NOTCH1 | NM_017617.3 |
| NOTCH2 | NM_024408.3 |
| NT5E | NM_002526.2 |
| PAX5 | NM_016734.1 |
| PDCD1 | NM_005018.1 |
| PDCD1LG2 | NM_025239.3 |
| PDCD2 | NM_144781.2 |
| PDGFB | NM_033016.2 |
| PDGFRB | NM_002609.3 |
| PECAM1 | NM_000442.3 |
| PIGR | NM_002644.2 |
| PLA2G2A | NM_000300.2 |
| PLA2G2E | NM_014589.1 |
| PLAU | NM_002658.2 |
| PLAUR | NM_001005376.1 |
| PML | NM_002675.3 |
| POU2F2 | NM_002698.2 |
| PPARG | NM_015869.3 |

| | |
|---|---|
| PPBP | NM_002704.2 |
| PRDM1 | NM_001198.3 |
| PRF1 | NM_005041.3 |
| PRKCD | NM_006254.3 |
| PSMB10 | NM_002801.2 |
| PSMB5 | NM_001130725.1 |
| PSMB7 | NM_002799.2 |
| PSMB8 | NM_004159.4 |
| PSMB9 | NM_002800.4 |
| PSMC2 | NM_002803.3 |
| PSMD7 | NM_002811.3 |
| PTAFR | NM_000952.3 |
| PTGER4 | NM_000958.2 |
| PTGS2 | NM_000963.1 |
| PTK2 | NM_005607.3 |
| PTPN2 | NM_002828.2 |
| PTPN22 | NM_015967.4 |
| PTPN6 | NM_002831.5 |
| PTPRC_all (comm | NM_080921.2 |
| CD45R0 | NM_080921.3 |
| CD45RA | NM_002838.4 |
| CD45RB | ENST00000367367.1 |
| PYCARD | NM_013258.3 |
| RAF1 | NM_002880.2 |
| RAG1 | NM_000448.2 |
| RAG2 | NM_000536.3 |
| RARRES3 | NM_004585.3 |
| RELA | NM_021975.2 |
| RELB | NM_006509.2 |
| RORC | NM_001001523.1 |
| RUNX1 | NM_001754.4 |
| S100A8 | NM_002964.3 |
| S100A9 | NM_002965.2 |
| S1PR1 | NM_001400.3 |
| SELE | NM_000450.2 |
| SELL | NR_029467.1 |
| SELPLG | NM_003006.3 |
| SERPING1 | NM_000062.2 |
| SH2D1A | NM_001114937.2 |
| SIGIRR | NM_021805.2 |
| SKI | NM_003036.2 |
| SLAMF1 | NM_003037.2 |
| SLAMF6 | NM_001184714.1 |
| SLAMF7 | NM_021181.3 |
| SLC2A1 | NM_006516.2 |
| SMAD3 | NM_005902.3 |

| | |
|---|---|
| SMAD5 | NM_005903.5 |
| SOCS1 | NM_003745.1 |
| SOCS3 | NM_003955.3 |
| SPP1 | NM_000582.2 |
| SRC | NM_005417.3 |
| STAT1 | NM_007315.2 |
| STAT2 | NM_005419.2 |
| STAT3 | NM_139276.2 |
| STAT4 | NM_003151.2 |
| STAT5A | NM_003152.2 |
| STAT5B | NM_012448.3 |
| STAT6 | NM_003153.3 |
| SYK | NM_003177.3 |
| TAGAP | NM_054114.3 |
| TAL1 | NM_003189.2 |
| TAP1 | NM_000593.5 |
| TAP2 | NM_000544.3 |
| TAPBP | NM_003190.4 |
| TBK1 | NM_013254.2 |
| TBX21 | NM_013351.1 |
| TCF4 | NM_003199.1 |
| TCF7 | NM_003202.2 |
| TFRC | NM_003234.1 |
| TGFB1 | NM_000660.3 |
| TGFBI | NM_000358.2 |
| TGFBR1 | NM_004612.2 |
| TGFBR2 | NM_001024847.1 |
| THY1 | NM_006288.2 |
| TICAM1 | NM_014261.1 |
| TIGIT | NM_173799.2 |
| TIRAP | NM_148910.2 |
| TLR1 | NM_003263.3 |
| TLR2 | NM_003264.3 |
| TLR3 | NM_003265.2 |
| TLR4 | NM_138554.2 |
| TLR5 | NM_003268.3 |
| TLR7 | NM_016562.3 |
| TLR8 | NM_016610.2 |
| TLR9 | NM_017442.2 |
| TMEM173 | NM_198282.1 |
| TNF | NM_000594.2 |
| TNFAIP3 | NM_006290.2 |
| TNFAIP6 | NM_007115.2 |
| TNFRSF10C | NM_003841.3 |
| TNFRSF11A | NM_003839.2 |
| TNFRSF13B | NM_012452.2 |

| | |
|---|---|
| TNFRSF13C | NM_052945.3 |
| TNFRSF14 | NM_003820.2 |
| TNFRSF17 | NM_001192.2 |
| TNFRSF1B | NM_001066.2 |
| TNFRSF4 | NM_003327.2 |
| TNFRSF8 | NM_152942.2 |
| TNFRSF9 | NM_001561.4 |
| TNFSF10 | NM_003810.2 |
| TNFSF11 | NM_003701.2 |
| TNFSF12 | NM_003809.2 |
| TNFSF13B | NM_006573.4 |
| TNFSF15 | NM_001204344.1 |
| TNFSF4 | NM_003326.2 |
| TNFSF8 | NM_001244.3 |
| TOLLIP | NM_019009.2 |
| TP53 | NM_000546.2 |
| TRAF1 | NM_005658.3 |
| TRAF2 | NM_021138.3 |
| TRAF3 | NM_145725.1 |
| TRAF4 | NM_004295.2 |
| TRAF5 | NM_004619.3 |
| TRAF6 | NM_145803.1 |
| TYK2 | NM_003331.3 |
| UBE2L3 | NM_198157.1 |
| VCAM1 | NM_001078.3 |
| VTN | NM_000638.3 |
| XBP1 | NM_005080.2 |
| XCL1 | NM_002995.1 |
| XCR1 | NM_005283.2 |
| ZAP70 | NM_001079.3 |
| ZBTB16 | NM_006006.4 |
| ZEB1 | NM_001128128.1 |
| **Internal Reference Genes** | |
| ABCF1 | NM_001090.2 |
| ALAS1 | NM_000688.4 |
| EEF1G | NM_001404.4 |
| G6PD | NM_000402.2 |
| GAPDH | NM_002046.3 |
| GUSB | NM_000181.1 |
| HPRT1 | NM_000194.1 |
| OAZ1 | NM_004152.2 |
| POLR1B | NM_019014.3 |
| POLR2A | NM_000937.2 |
| PPIA | NM_021130.2 |
| SDHA | NM_004168.1 |
| TBP | NM_001172085.1 |

| | |
|---|---|
| TUBB | NM_178014.2 |
| RPL19 | NM_000981.3 |

**Custome code set probes**

| **Official Symbol** | **Accession** |
|---|---|
| SIGLEC1 | NM_023068.3 |
| BLK | NM_001715.2 |
| VH4-34 (9G4) | AY058025.1 |
| PARP9 | NM_031458.2 |
| BANK1 | NM_017935.4 |
| TNFRSF13C | NM_052945.3 |
| NRP4 | NM_003873.5 |
| IRF9 | NM_006084.4 |
| TNFSF10 (TRAIL) | NM_003810.3 |
| HAVCR1 | NM_012206.3 |
| RASGRP3 | NM_001139488.1 |
| SKAP2 | NM_003930.4 |
| BACH2 | NM_021813.3 |
| PTPN11 | NM_002834.3 |
| PLCG1 | NM_002660.2 |
| CD300a | NM_007261.3 |
| NCR2 | NM_004828.3 |
| PLXNA1 | NM_032242.3 |
| TYROBP | NM_003332.3 |
| S1PR4 | NM_003775.3 |
