# Supplementary material for: Plasmacytoid dendritic cells and RNA-containing immune complexes drive expansion of peripheral B cell subsets with an SLE-like phenotype
Source: PLoS One. 2017 Aug 28;12(8):e0183946. doi: 10.1371/journal.pone.0183946 (PMC5573130; doi:10.1371/journal.pone.0183946)
Supplement: S2 Table — (PDF) [file pone.0183946.s006.pdf]

## Supplementary Table S2

Median values (n=10) of gene counts (nCounter)

| Gene name | Median value CD27-IgD- B cells | Median value CD27+IgD- B cells |
|---|---|---|
| CD74 | 35908,9 | 34650,1 |
| HLA-B | 18297,4 | 18033,0 |
| B2M | 16931,7 | 20686,9 |
| HLA-A | 12308,1 | 11375,5 |
| MX1 | 9600,5 | 11776,7 |
| HLA-DRA | 8924,1 | 11051,0 |
| IFITM1 | 6695,0 | 7798,4 |
| HLA-C | 6478,2 | 4597,9 |
| HLA-DRB3 | 4736,5 | 4589,6 |
| CXCR4 | 4326,2 | 3087,0 |
| MS4A1 | 3898,4 | 5551,7 |
| STAT1 | 3788,6 | 4110,8 |
| STAT2 | 3450,5 | 3015,5 |
| CD79A | 3401,4 | 3681,8 |
| HLA-DPA1 | 3210,3 | 4203,3 |
| BANK1 | 2955,3 | 4871,9 |
| STAT6 | 2931,6 | 2802,5 |
| PTPRC_all | 2657,3 | 3593,7 |
| IRF7 | 2536,8 | 2590,6 |
| CD83 | 2280,5 | 1161,3 |
| IFIT2 | 2254,5 | 2853,5 |
| CCL4 | 2176,5 | 522,3 |
| CD48 | 2168,9 | 2198,0 |
| PML | 2083,6 | 1962,7 |
| ARHGDIB | 1981,5 | 2340,3 |
| IFI16 | 1953,7 | 2227,0 |
| TNFSF10 | 1897,6 | 1892,0 |
| HLA-DPB1 | 1778,6 | 2361,9 |
| CCR7 | 1750,6 | 1016,4 |
| MCL1 | 1732,7 | 2299,3 |
| PSMB9 | 1728,4 | 1900,4 |
| CD79B | 1710,8 | 2131,3 |
| CD53 | 1695,0 | 2497,6 |
| PARP9 | 1523,7 | 1734,0 |
| CD19 | 1485,8 | 1887,0 |
| IRF8 | 1400,9 | 1017,7 |
| SELL | 1367,4 | 1526,7 |
| TNFRSF13C | 1319,7 | 2084,4 |
| PSMB8 | 1240,7 | 1331,5 |
| TAP1 | 1217,9 | 1263,0 |

| | | |
|---|---|---|
| ILF3 | 1184,8 | 1143,3 |
| IL2RG | 1181,2 | 1083,5 |
| HLA-DMB | 1124,7 | 1315,4 |
| BTLA | 1120,4 | 589,6 |
| IL32 | 1099,8 | 1233,4 |
| BCL2 | 1089,7 | 1025,6 |
| CD22 | 1081,4 | 1174,2 |
| IRF9 | 1069,3 | 1083,5 |
| JAK2 | 1039,7 | 1007,0 |
| TGFBR2 | 1038,6 | 834,9 |
| BAX | 1015,8 | 951,5 |
| PSMB10 | 1009,5 | 964,1 |
| IRF4 | 952,6 | 661,3 |
| RASGRP3 | 929,9 | 867,1 |
| HLA-DMA | 923,3 | 961,1 |
| CD45RA | 920,5 | 1627,8 |
| MAP4K4 | 910,5 | 800,0 |
| CTSS | 879,2 | 1038,2 |
| PTPN6 | 869,1 | 753,0 |
| TAP2 | 854,8 | 781,2 |
| TAPBP | 830,1 | 975,4 |
| IFIH1 | 825,2 | 827,8 |
| NFKB2 | 811,3 | 507,3 |
| CD81 | 792,9 | 945,3 |
| IRF1 | 786,1 | 785,2 |
| CTNNB1 | 755,9 | 784,4 |
| JAK1 | 754,7 | 916,6 |
| MIF | 753,1 | 569,1 |
| HLA-DQA1 | 739,7 | 899,0 |
| STAT3 | 730,2 | 805,7 |
| GBP1 | 717,7 | 651,9 |
| MYD88 | 706,4 | 772,9 |
| TGFB1 | 706,0 | 727,6 |
| CD44 | 695,7 | 922,1 |
| SOCS1 | 674,8 | 764,8 |
| PTPN1 | 673,5 | 971,4 |
| IKZF3 | 668,8 | 1484,0 |
| NFKBIA | 657,6 | 439,6 |
| PAX5 | 646,5 | 649,8 |
| TP53 | 628,3 | 563,3 |
| TCF4 | 612,7 | 929,1 |
| C14orf166 | 607,6 | 557,0 |
| CD40 | 598,8 | 620,8 |
| ITGA4 | 588,6 | 538,7 |
| VH4-34 | 586,7 | 228,6 |
| IFI35 | 579,0 | 528,7 |

| | | |
|---|---|---|
| IL16 | 536,0 | 643,0 |
| BST2 | 529,7 | 639,4 |
| POU2F2 | 523,3 | 701,2 |
| GPI | 513,4 | 352,0 |
| CD24 | 510,8 | 1503,4 |
| MAPK1 | 501,9 | 497,9 |
| IL4R | 500,9 | 105,4 |
| C1QBP | 500,8 | 436,0 |
| HLA-DOB | 498,5 | 723,3 |
| CD164 | 478,3 | 432,2 |
| BLNK | 477,7 | 504,0 |
| BLK | 467,8 | 786,3 |
| NOTCH2 | 465,1 | 458,7 |
| CD46 | 449,9 | 492,6 |
| LAMP3 | 449,7 | 224,0 |
| ICAM3 | 430,5 | 573,6 |
| SLAMF1 | 428,2 | 200,4 |
| FCGR2B | 425,3 | 673,8 |
| CASP8 | 412,4 | 487,4 |
| TLR7 | 411,8 | 389,6 |
| IFNAR2 | 409,8 | 457,7 |
| MALT1 | 403,0 | 355,4 |
| ETS1 | 400,4 | 513,3 |
| IL10RA | 400,3 | 685,3 |
| CD99 | 395,9 | 466,8 |
| LITAF | 395,5 | 271,6 |
| SMAD3 | 385,6 | 258,4 |
| BCL10 | 384,4 | 415,0 |
| SKAP2 | 373,6 | 499,5 |
| SYK | 372,3 | 579,2 |
| IKZF2 | 367,1 | 36,9 |
| MAP4K2 | 360,4 | 372,5 |
| TAGAP | 359,5 | 417,6 |
| RELA | 358,3 | 326,3 |
| CASP2 | 345,3 | 342,2 |
| MAPKAPK2 | 344,5 | 323,5 |
| BCAP31 | 343,5 | 341,9 |
| BTK | 341,9 | 375,1 |
| TBK1 | 341,4 | 417,2 |
| PSMD7 | 331,3 | 309,5 |
| IRAK1 | 318,5 | 283,3 |
| CCR6 | 317,6 | 424,1 |
| PSMB7 | 311,1 | 272,7 |
| PTPN11 | 310,1 | 284,5 |
| MAPK14 | 305,4 | 248,1 |
| PTPN2 | 291,6 | 345,7 |

| | | |
|---|---|---|
| MAP4K1 | 277,3 | 460,8 |
| TNFSF13B | 274,7 | 240,9 |
| CD97 | 273,5 | 291,9 |
| NFATC1 | 268,5 | 221,2 |
| IRF5 | 266,8 | 242,3 |
| S1PR1 | 265,8 | 270,5 |
| NFATC3 | 265,3 | 209,7 |
| TRAF4 | 265,0 | 244,8 |
| LAG3 | 264,1 | 216,4 |
| IFNGR1 | 263,6 | 264,5 |
| CCL22 | 263,2 | 144,0 |
| TNFAIP3 | 262,6 | 190,7 |
| CYBB | 256,6 | 360,3 |
| BACH2 | 256,3 | 157,2 |
| IL6ST | 252,6 | 181,9 |
| CCND3 | 252,3 | 149,6 |
| TLR1 | 250,9 | 330,8 |
| ICAM2 | 249,2 | 111,6 |
| PSMB5 | 248,1 | 196,6 |
| CXCL10 | 247,9 | 216,5 |
| NCF4 | 246,0 | 455,5 |
| GBP5 | 244,3 | 248,4 |
| TYK2 | 243,6 | 272,3 |
| CCL3 | 243,1 | 106,9 |
| IL13RA1 | 238,9 | 143,0 |
| LTA | 237,4 | 171,3 |
| TRAF5 | 235,8 | 393,8 |
| PRKCD | 232,7 | 341,4 |
| CD59 | 229,2 | 226,9 |
| TRAF2 | 228,5 | 224,0 |
| PECAM1 | 227,6 | 315,7 |
| JAK3 | 221,1 | 284,3 |
| NFATC2 | 219,6 | 286,4 |
| CHUK | 215,7 | 199,1 |
| CD58 | 214,2 | 225,8 |
| TNF | 211,3 | 283,8 |
| PDCD2 | 207,5 | 188,7 |
| IKBKB | 205,0 | 228,7 |
| TNFRSF14 | 201,3 | 211,8 |
| TRAF3 | 200,0 | 267,7 |
| TFRC | 197,3 | 247,5 |
| NFKB1 | 190,9 | 125,1 |
| RAF1 | 184,6 | 232,5 |
| HLA-DQB1 | 179,1 | 194,7 |
| LILRB1 | 176,5 | 165,1 |
| IKBKG | 176,2 | 170,0 |

| | | |
|---|---|---|
| CIITA | 175,9 | 188,8 |
| GPR183 | 175,6 | 571,2 |
| CASP1 | 175,6 | 290,8 |
| ITGB1 | 174,5 | 487,7 |
| STAT5A | 172,7 | 168,6 |
| NFKBIZ | 172,0 | 261,4 |
| BCL3 | 166,1 | 206,9 |
| CUL9 | 165,2 | 190,6 |
| IRAK4 | 164,7 | 188,9 |
| TRAF1 | 163,0 | 104,8 |
| TNFRSF17 | 162,9 | 188,5 |
| PTK2 | 161,6 | 193,0 |
| IL21R | 154,7 | 33,4 |
| ENTPD1 | 153,8 | 160,4 |
| FYN | 153,6 | 153,3 |
| CD45RB | 152,1 | 82,3 |
| CTSC | 151,6 | 179,1 |
| ITGB2 | 151,2 | 98,9 |
| STAT5B | 147,8 | 219,1 |
| LCK | 146,9 | 143,7 |
| IGF2R | 142,4 | 110,9 |
| APP | 142,3 | 105,3 |
| ICOSLG | 142,0 | 59,0 |
| PSMC2 | 141,4 | 157,9 |
| NT5E | 141,4 | 89,9 |
| IKBKE | 141,2 | 238,6 |
| ITGAE | 141,1 | 109,4 |
| TICAM1 | 138,8 | 154,2 |
| NOD1 | 136,4 | 141,4 |
| STAT4 | 134,3 | 99,5 |
| CASP3 | 131,4 | 104,3 |
| TNFRSF1B | 130,5 | 165,5 |
| BCL6 | 127,9 | 101,1 |
| CD70 | 127,0 | 262,8 |
| IRF3 | 126,7 | 141,6 |
| TRAF6 | 126,7 | 121,9 |
| TGFBR1 | 125,3 | 139,6 |
| BATF3 | 124,8 | 56,8 |
| FKBP5 | 124,3 | 125,0 |
| TNFRSF13B | 122,8 | 832,9 |
| XBP1 | 119,8 | 205,7 |
| IL15 | 119,7 | 116,5 |
| TLR9 | 117,6 | 279,0 |
| ZEB1 | 116,7 | 157,1 |
| IKZF1 | 114,2 | 167,6 |
| ATG16L1 | 113,2 | 109,9 |

| | | |
|---|---|---|
| IL12RB1 | 110,6 | 122,4 |
| TOLLIP | 110,4 | 111,9 |
| ITGAL | 109,1 | 193,4 |
| RELB | 101,5 | 87,3 |
| ATG5 | 100,8 | 121,6 |
| BCL2L11 | 100,3 | 93,0 |
| SKI | 97,7 | 132,5 |
| UBE2L3 | 96,1 | 99,4 |
| RARRES3 | 94,8 | 80,6 |
| PTGER4 | 93,0 | 65,6 |
| PLCG1 | 92,8 | 136,2 |
| THADA | 92,0 | 106,1 |
| SLAMF6 | 90,9 | 65,6 |
| SLAMF7 | 90,6 | 121,3 |
| ATG7 | 87,8 | 97,4 |
| CSF2RB | 85,1 | 133,5 |
| RUNX1 | 84,6 | 59,2 |
| ABCB1 | 82,6 | 30,2 |
| GZMB | 81,6 | 27,5 |
| FCGRT | 80,2 | 87,8 |
| BID | 79,7 | 55,3 |
| CD96 | 79,5 | 52,7 |
| SMAD5 | 79,4 | 96,0 |
| S1PR4 | 79,4 | 99,6 |
| ABL1 | 78,3 | 82,1 |
| TNFSF12 | 76,6 | 112,8 |
| NOTCH1 | 74,9 | 77,9 |
| CD82 | 68,7 | 129,9 |
| SRC | 68,6 | 86,0 |
| MBP | 68,0 | 98,6 |
| CD274 | 66,6 | 65,9 |
| BATF | 65,9 | 96,0 |
| LAIR1 | 64,2 | 33,9 |
| CD80 | 60,7 | 127,6 |
| AHR | 59,9 | 78,7 |
| IKBKAP | 59,6 | 51,3 |
| LCP2 | 57,9 | 49,7 |
| ATG12 | 56,4 | 51,5 |
| CDKN1A | 55,3 | 58,5 |
| TCF7 | 55,2 | 178,8 |
| LY96 | 55,0 | 91,4 |
| TBX21 | 54,8 | 69,3 |
| CXCR3 | 53,4 | 108,2 |
| NOD2 | 52,9 | 74,8 |
| PYCARD | 52,8 | 85,4 |
| ICAM1 | 52,5 | 56,5 |

| | | |
|---|---|---|
| CD86 | 52,0 | 95,6 |
| ATG10 | 50,5 | 48,6 |
| EGR1 | 50,3 | 62,8 |
| IRAK2 | 48,8 | 60,0 |
| MR1 | 44,3 | 55,6 |
| SIGIRR | 43,9 | 53,9 |
| ADA | 41,3 | 92,0 |
| IDO1 | 40,9 | 60,7 |
| CCR1 | 38,4 | 119,1 |
| CD300a | 35,8 | 80,9 |
| PRDM1 | 32,9 | 90,2 |
| IL2RB | 28,0 | 51,7 |
| CEACAM1 | 27,8 | 55,2 |
| CD6 | 27,2 | 51,2 |
| IL2RA | 21,0 | 87,3 |
| FCGR2A/C | 20,8 | 106,1 |
| CD27 | 18,4 | 688,0 |
| HLA-DRB1 | 8,9 | 153,7 |
